# Effects of vitamins and polyunsaturated fatty acids on cognitive function in older adults with mild cognitive impairment: a meta-analysis of randomized controlled trials

**DOI:** 10.1007/s00394-024-03324-y

**Published:** 2024-02-01

**Authors:** Jing Chang, Minhui Liu, Chang Liu, Shiyu Zhou, Yuchen Jiao, Hongyu Sun, Yan Ji

**Affiliations:** 1 School of Nursing, Nanjing Medical University, 101 Longmian Avenue, Jiangning District, Nanjing, 211166 Jiangsu China; 2 School of Nursing, Ningxia Medical University, 1160 Shengli South Street of Xingqing District, Yinchuan, 750001 China; 3 School of Nursing, Peking University, 38 College Road, Haidian District, Beijing, 100191 China

**Keywords:** Cognitive function, Meta-analysis, Mild cognitive impairment, Polyunsaturated fatty acids, Vitamins

## Abstract

**Purpose:**

Vitamins and polyunsaturated fatty acids (PUFAs) have been studied extensively as safe and manageable nutrient interventions for mild cognitive impairment (MCI). The purpose of the current meta-analysis was to examine the effects of vitamins and PUFAs on cognition and to compare the effects of single and multiple nutrient subgroups in patients with MCI.

**Methods:**

Randomized controlled trials (RCTs) written in English and Chinese were retrieved from eight databases, namely, PubMed, CENTRAL, Embase, CINAHL, Web of Science, SinoMed, CNKI, and Wanfang Data, from their respective dates of inception until 16 July 2023. The quality of the included studies was assessed using the Cochrane Risk of Bias Tool 2.0. Meta-analyses were performed to determine the standardized mean differences (SMDs) in global cognitive function, memory function, attention, visuospatial skills, executive function, and processing speed between the supplement and control groups using 95% confidence intervals (CI) and *I*^2^. Prospero registration number: CRD42021292360.

**Results:**

Sixteen RCTs that studied different types of vitamins and PUFAs were included. The meta-analysis revealed that vitamins affected global cognitive function (SMD = 0.58, 95% CI = [0.20, 0.96], *P* = 0.003), memory function (SMD = 2.55, 95% CI = [1.01, 4.09], *P* = 0.001), and attention (SMD = 3.14, 95% CI = [1.00, 5.28], *P* = 0.004) in patients with MCI, and PUFAs showed effects on memory function (SMD = 0.65, 95% CI = [0.32, 0.99], *P* < 0.001) and attention (SMD = 2.98, 95% CI = [2.11, 3.84], *P* < 0.001). Single vitamin B (folic acid [FA]: SMD = 1.21, 95% CI = [0.87, 1.55]) supplementation may be more effective than multiple nutrients (FA and vitamin B12: SMD = 0.71, 95% CI = [0.41, 1.01]; and FA combined with docosahexaenoic acid [DHA]: SMD = 0.58, 95% CI = [0.34, 0.83]) in global cognitive function.

**Conclusions:**

FA, vitamin B6, vitamin B12, and vitamin D may improve global cognitive function, memory function, and attention in patients with MCI. Eicosapentaenoic acid (EPA) and DHA may improve memory function and attention. We also noted that FA may exert a greater effect than a vitamin B combination (FA and vitamin B12) or the combination of FA and DHA. However, because of the low evidence-based intensity, further trials are necessary to confirm these findings.

**Supplementary Information:**

The online version contains supplementary material available at 10.1007/s00394-024-03324-y.

## Introduction

The construct of mild cognitive impairment (MCI) emphasizes global cognitive dysfunction and various degrees of impairment within cognitive domains, including memory, attention, visuospatial skills, executive function, and processing speed [1, 2]. Patients with MCI experience an increasing risk of progressing to dementia as their condition worsens [3, 4]. Individuals with MCI aged ≥ 65 years exhibited a high 2-years dementia incidence (14.9%) [5]. Therefore, effective interventions to mitigate cognitive impairment and prevent dementia are urgently needed [6].

Various interventions have been implemented in patients with MCI to improve function in various cognitive domains and delay dementia [7, 8]. Nutrients rank highly among these interventions, as they are less costly and have an improved safety profile [9, 10]. Among the micronutrients, vitamin B (vitamin B6, vitamin B12, or folic acid [FA]) can reduce high homocysteine concentrations, which are related to brain health [11, 12]. Vitamin D, C, or E have anti-inflammatory and neuroprotective effects [11, 12]. Among the macronutrients, *n *− 3 polyunsaturated fatty acids (PUFAs) (eicosapentaenoic acid [EPA] or docosahexaenoic acid [DHA]), lipidic molecules with several double bonds in their aliphatic chains, are linked to a reduced risk of cognitive decline [11, 13]. In addition, multiple nutrients of the same (complex supplements of FA, vitamin B12, and/or vitamin B6, or complex supplements of EPA and DHA) and different categories (vitamins combined with PUFAs) have also been explored for their potential effects on cognition [14, 15].

Although numerous systematic reviews have examined the effects of vitamins and polyunsaturated fatty acids (PUFAs) on cognitive function, their findings have been inconsistent, likely due to variability in factors such as research samples and study designs [16, 17]. For instance, divergent outcomes have arisen in the analysis of Vitamin B’s impact across different cognitive domains, which underscores the current lack of robust data to establish a definitive causal relationship between nutrients and cognitive function [17, 18]. Consequently, there remains a dearth of conclusive recommendations for using vitamins and PUFAs to address mild cognitive impairment (MCI).

Comparative analyses of single versus combined nutrient interventions are sparse. Notwithstanding, recent trials have begun to assess these differences. For example, a randomized controlled trial (RCT) indicated that a combination of folic acid (FA) and vitamin B12 was more efficacious than either nutrient alone [19]. Similarly, RCTs in 2021 found the pairing of FA and docosahexaenoic acid (DHA) to be superior in cognitive enhancement than singular nutrient administration [20, 21]. However, these trials were limited by small sample sizes and short follow-up periods, which may not fully capture the cognitive effects. As such, meta-analyses are indispensable for deriving stronger evidence.

In summary, this meta-analysis aimed to (1) systematically summarize existing RCTs to assess the effects of vitamins and PUFAs and (2) perform a comparative analysis of the effect of single and multiple nutrients on global cognitive function and various cognitive domains, including memory function, attention, visuospatial skills, executive function, and processing speed, in patients with MCI.

## Materials and methods

### Design

This meta-analysis of RCTs was based on the Preferred Reporting Items for Systematic Reviews and Meta-Analysis checklist [22]. The study was registered in the PROSPERO register (CRD42021292360) and is available at http://www.crd.york.ac.uk/PROSPERO.

### Eligibility criteria

Studies that met the following criteria were included: (1) patients aged ≥ 60 years were diagnosed with MCI using the modified Petersen’s diagnostic standard [23] (participant); (2) the interventions consisted of vitamins and/or PUFAs taken orally (intervention); (3) control interventions had no specific risk-modifying effects (e.g., usual care or placebo) (comparison); (4) the outcomes included the following: effect on global cognitive function and/or cognitive domains (memory, attention, visuospatial skills, executive function, and processing speed) (outcome); (5) RCTs (study design).

Studies that met the following criteria were excluded: (1) patients with psychiatric problems (e.g., depression) or comorbid conditions that may alter performance on cognitive tests (e.g., stroke, head injuries, Parkinson’s disease, and learning disabilities); (2) cognitive interventions, physical interventions, or drug interventions; (3) pilot trials denoted in the title or abstract; (4) narrative or systematic reviews, conference abstracts, and protocols; (5) reports not written in English or Chinese; (6) animal experiments; and (7) duplicated publications.

### Search strategy

We searched the following databases from their dates of inception to July 16, 2023, for relevant studies in English and Chinese: PubMed, the Cochrane Central Register of Controlled Trials (CENTRAL), Excerpta Medica Database (EMBASE), Cumulative Index to Nursing and Allied Health Literature (CINAHL), Web of Science, the Chinese Bio-Medical Literature Database (SinoMed), China National Knowledge Infrastructure (CNKI), and Wanfang Data. The search string was built as follows: (mild cognitive impairment OR cognitive dysfunction) AND (nutrient OR vitamin OR fatty acid) AND (randomized controlled trials). The full search strategy used for the eight databases is shown in Supplementary Table S1. The electronic database search was supplemented by a manual search of the reference lists of the articles included for potentially related articles.

### Study selection and data extraction

Two reviewers (Jing Chang and Shiyu Zhou) independently identified studies that met the inclusion criteria by screening the titles and abstracts. If the information in the title and abstract was insufficient, the full text was obtained to determine the eligibility of the study for inclusion in this meta-analysis. If numerous articles were written about an RCT, the article that reported the highest number of outcomes was included. Any disagreements were resolved after discussions with three other reviewers (Hongyu Sun, Yan Ji, and Minhui Liu). For each eligible study, information about the first author’s name, publication year, country, diagnostic criteria for MCI, characteristics of participants, intervention duration, supplementation doses, outcome measures, baseline blood concentrations of nutrients, and baseline cognition of participants was extracted using a self-designed standardized form. We extracted the long-term data (e.g., twelve-month data) reported by the study if the outcome data were available at different time points (e.g., 3, 6, and 12 months). If a trial had two or more nutrient groups, the sample size, mean, and standard deviation of each group were pooled into a single measurement [24]. In addition, the mean, standard deviation, and sample size of the intervention group and control group of each trial were extracted for meta-analysis. We excluded trials from the meta-analysis in which the mean, standard deviation, and sample size could not be obtained despite our best attempts. Two reviewers (Jing Chang and Shiyu Zhou) independently extracted the data using EndNote X9.3.3.

### Risk of bias assessment

Two reviewers (Jing Chang and Shiyu Zhou) used the Cochrane risk-of-bias tool 2.0 [25] to independently assess the risk of bias in the included RCTs. The key assessment areas included bias arising from the randomization process, deviations from intended interventions, due to missing outcome data, in the measurement of the outcome, and in the selection of the reported results. Each criterion was qualified as either low, some concerns, or a high risk of bias. The overall quality of each study was determined by checking each criterion for the five domains of the risk of bias. Disagreements between the reviewers were resolved by consensus through discussion with three other expert reviewers (Hongyu Sun, Yan Ji, and Minhui Liu).

### Outcome measurements

The primary outcome of interest was the global cognitive function level, which was measured using the mini-mental state examination (MMSE), full-scale intelligence quotient (FSIQ), basic cognitive aptitude tests (BCAT), clinical dementia rating scale sum of boxes (CDR-SOB), or the repeatable battery for the assessment of neuropsychological status (RBANS). The secondary outcomes were memory function, measured with the Hopkins Verbal Learning Test-revised (HVLT-R), the digit span from FSIQ, or Rey Auditory Verbal Learning Test (RAVLT); attention, measured using the Trail Making Test A (TMT-A), the digit span from FSIQ, or symbol cancelation test; visuospatial skills, measured using the block design from FSIQ; executive function, measured with the trail making test B (TMT-B), clock drawing test (CDT), or executive clock drawing task (CLOX); processing speed, measured with the TMT-A or the digit symbol from FSIQ.

### Data synthesis and meta-analysis

Review Manager 5.3 software (The Nordic Cochrane Centre, The Cochrane Collaboration, Copenhagen, Denmark) was used to perform the meta-analysis. Because all data were continuous, the data were expressed using the mean difference (MD) with 95% confidence intervals (CIs). The standardized mean difference (SMD) was used when different scales were applied to measure the same outcome. Heterogeneity in the included studies was quantified using the I2 statistic. When the *I*^2^ was > 60%, the study was considered to show high heterogeneity. Sensitivity analysis was used to analyze the source of heterogeneity by excluding studies of poor quality or those in which the control interventions potentially exerted potential treatment effects. We also carried out subgroup analyses to identify potential sources of heterogeneity, which we based on probable covariates: the number of participants within each group, the ages of participants, and the duration of the supplement intervention. For instances where more than two subgroups existed, meta-regression was employed to investigate the interaction effect. The type of subgroup analysis was prespecified to determine whether the summary effects varied with the clinical characteristics of the included trials. We performed subgroup analysis according to the different types of vitamins and PUFAs to compare different treatment effects between single and multiple nutrients. The SMD determines the significance of the pooled effect size. Publication bias was assessed using funnel plots, which visually represented the estimation of the treatment effect in the studies included in the meta-analysis. If publication bias exists, the funnel plot is affected by an asymmetrical appearance, and the meta-analysis could overestimate the treatment effect. The “metafunnel” package was adopted to generate the funnel plot in Stata MP, version 14.0 software.

## Results

### Study selection

**Fig. 1:**
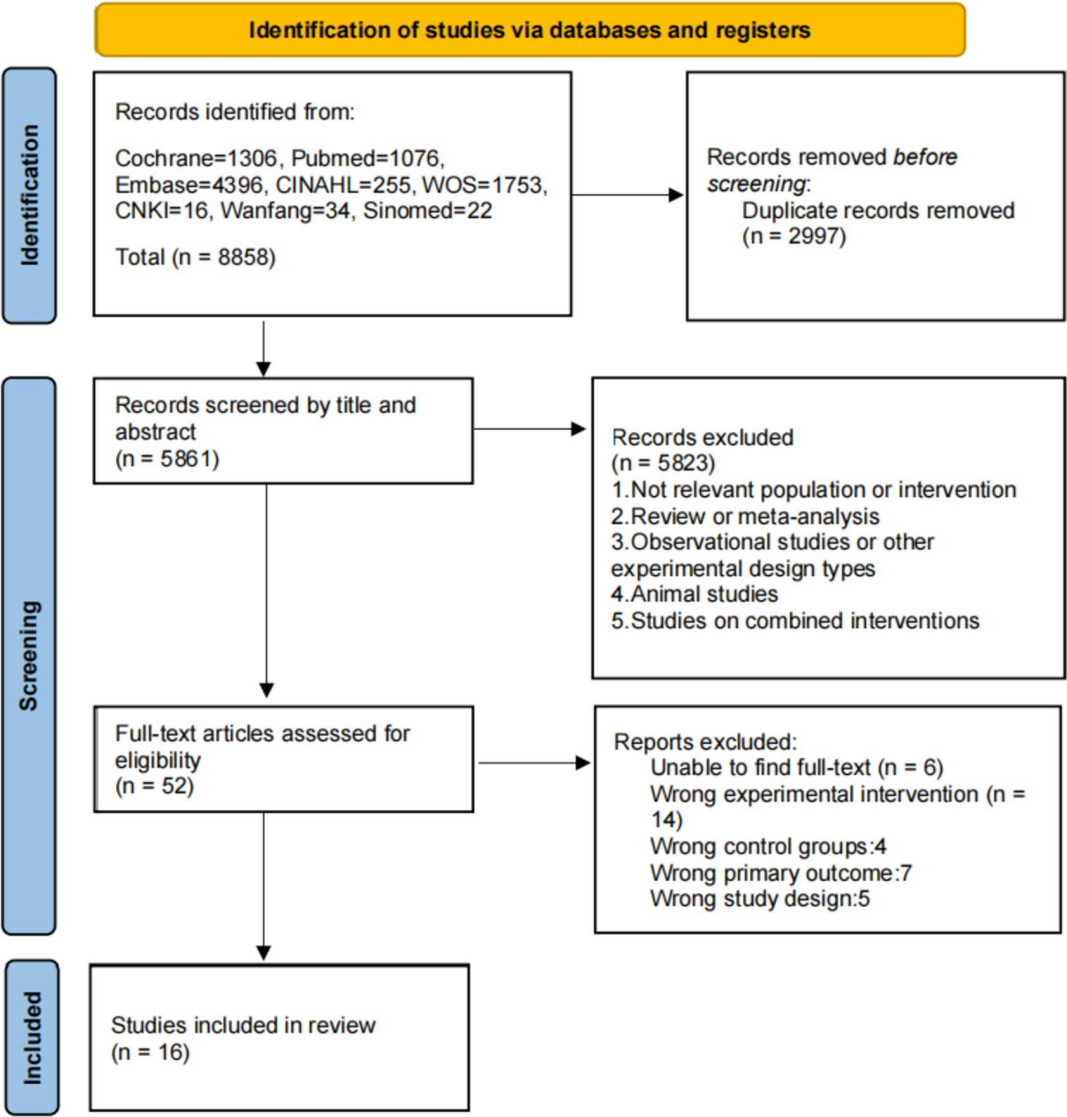
Flowchart of the search results in the review

Initially, 8858 potential articles were retrieved from the electronic databases and reference lists. After removing duplicates, 5861 articles were screened based on their titles and abstracts. Five thousand eight hundred and twenty-three articles were excluded because they did not meet the inclusion criteria. We then assessed the 52 remaining articles for eligibility based on the full text, and 16 RCTs [19–21, 26–38] were included in this meta-analysis. A flow diagram of the selection procedure is shown in Fig. 1.

### Characteristics of the included studies

The study’s characteristics are listed in Table 1. Sixteen included studies were published between 2012 and 2022. The studies were mainly conducted in China (*n* = 10), and the rest were conducted in New Zealand (*n* = 1), the United Kingdom (*n* = 2), Cyprus (*n* = 1), Iran (*n* = 1), and Malaysia (*n* = 1). The studies identified 2528 participants, with a sample size of 30–256. One thousand four hundred forty-five were female, representing 57.2% of the total participants. The mean age of the participants in the intervention group ranged from 65.5 to 77.6, whereas the mean age in the control group ranged from 63.5 to 81.2. There were no significant differences in blood concentrations of nutrients or cognitive function between participants in the intervention group and control group.

**Table 1:** Characteristics of the included studies

Author (year)	Country	Diagnostic criteria for MCI	Characteristics of Participants			Type of nutrients	Duration	Outcome measures	Baseline blood concentrations of nutrients (IG/CG)	Baseline global cognition of participants (IG/CG)
			N (IG/CG)	Mean age (years) (IG/CG)	Women (*n*/%) (IG/CG)					
Mengelberg et al. [38]	New Zealand	Petersen criteria 2004 [23]	30/30	72.3 ± 6.16/73.4 ± 6.96	16(53)/19(63)	1491 mg DHA and 351 mg EPA	12 months	1. Global cognition: RBANS 2. Memory: digit span 3. Executive function: TMT-B	DHA (%/TFA): 4.1(1.01)/4.06(0.96)	RBANS: 101.9 ± 11.9/100.3 ± 11.9
Perła-Kaján et al. [30]	United Kingdom	Petersen criteria 2004 [23]	95/101	Total: 77.6 ± 4.8	Total: 118(60.0)	0.8 mg FA, 0.5 mg vitamin B_12_ and 20 mg vitamin B_6_	2 years	1. Global cognition: MMSE 2. Memory: HVLT-R 3. Attention: TMT-A 4. Processing speed: TMT-A 5. Executive function: TMT-B	NA	MMSE: 28.1 ± 1.8/28.2 ± 1.5
Li et al. [16]	China	Petersen criteria 2004 [23]	FA + DHA group (*n* = 60) FA group (*n* = 60) DHA group (*n* = 60) Placebo group (*n* = 60)	FA + DHA group: 70.3 ± 7.7 FA group: 70.2 ± 6.1 DHA group: 71.6 ± 6.6 Placebo group: 70.4 ± 6.7	FA + DHA group: 36(60.0) FA group: 36(60.0) DHA group: 36(60.0) Placebo group: 33(55.0)	800 μg FA and 800 mg DHA	6 months	1. Global cognition: FSIQ 2. Memory: digit span 3. Visuospatial skills: block design 4. Attention: digit span	FA (ng/ml): FA + DHA group: 7.46 ± 3.01 FA group: 8.41 ± 4.15 DHA group: 8.25 ± 3.96 Placebo group: 7.99 ± 4.84 DHA (μg/ml): FA + DHA group: 18.64 ± 6.79 FA group: 17.41 ± 4.17 DHA group: 19.19 ± 4.77 Placebo group: 18.30 ± 6.54	MMSE: FA + DHA group: 20.40 ± 2.57 FA group: 21.02 ± 2.15 DHA group: 21.25 ± 2.35 Placebo group: 21.33 ± 1.90
Bai et al. [20]	China	Petersen criteria 2004 [23]	FA + DHA (*n* = 34) FA (*n* = 35) DHA (*n* = 36) Placebo (*n* = 33)	FA + DHA: 66.7 ± 5.8 FA: 67.5 ± 5.1 DHA: 70.2 ± 6.5 Placebo: 68.3 ± 6.4	FA + DHA: 20 (58.8) FA: 23 (65.7) DHA: 25 (69.4) Placebo: 19 (57.6)	0.8 mg FA and 800 mg DHA	6 months	1. Global cognition: FSIQ 2. Memory: digit span 3. Visuospatial skills: block design 4. Attention: digit span	FA (ng/ml): FA + DHA group: 6.97 ± 2.24 FA group: 8.30 ± 3.84 DHA group: 8.28 ± 4.04 Placebo group: 9.02 ± 5.90 DHA (μg/ml): FA + DHA group: 19.05 ± 8.65 FA group: 17.47 ± 6.58 DHA group: 19.36 ± 5.96 Placebo group: 17.58 ± 8.12	MMSE: FA + DHA group: 20.22 ± 3.21 FA group: 20.44 ± 2.36 DHA group: 20.54 ± 2.63 Placebo group: 21.30 ± 2.15
Kwok et al. [37]	China	Petersen criteria 2004 [23]	138/141	76.9 ± 5.4/78.0 ± 5.3	51(36.9)/62(43.9)	400 μg FA and 500 μg vitamin B_12_	24 months	1. Global cognition: CDR_SOB 2. Memory: CDR 3. Executive function: CDR	FA (nmol/L): 27.8 ± 8.0/29.4 ± 8.6	MMSE: 26.0 ± 3.1/25.7 ± 3.0
Yang et al. [31]	China	Petersen criteria 2004 [23]	93/90	67.2 ± 6.1/66.6 ± 5.2	50(53.8)/51(56.7)	800 IU Vitamin D	12 months	1. Global cognition: FSIQ 2. Memory: digit span 3. Visuospatial skills: block design 4. Attention: digit span 5. Processing speed: digit symbol	Vitamin D (ng/mL): 19.07 ± 2.91/19.78 ± 2.88	MMSE: 22.76 ± 2.02/22.40 ± 1.89
Stavrinou et al. [36]	Cyprus	Petersen criteria 2004 [23]	18/18	77.4 ± 9.2/81.2 ± 5.3	10(55.6)/12(66.7)	*n* − 3 (810 mg EPA, 4140 mg DHA), * n* − 6 (1800 mg GLA, 3150 mg LA), 0.6 mg vitamin A, vitamin E (22 mg α-tocopherol and 760 mg pureγ-tocopherol)	6 months	1. Global cognition: MMSE 2. Processing speed: TMT-A 3. Executive function: TMT-B 4. Attention: a symbol cancelation test	NA	MMSE: 24.3 ± 3.5/23.6 ± 3.2
Ma et al. [28]	China	Petersen criteria 2004 [23]	90/90	74.8 ± 2.8/74.6 ± 3.2	51(56.67)/52(57.78)	400 μg FA	24 months	1. Global cognition: FSIQ 2. Memory: digit span 3. Visuospatial skills: block design 4. Attention: digit span 5. Processing speed: digit symbol	FA (ng/mL): 7.01 ± 0.64/5.79 ± 0.67	MMSE: 21.33 ± 1.56/20.99 ± 1.06
Ma et al. [19]	China	Petersen criteria 2004 [23]	FA + vitamin B_12_ group (*n* = 60) FA group (*n* = 60) Vitamin B_12_ group (*n* = 60) Placebo group (*n* = 60)	FA + vitamin B_12_ group: 69.2 ± 2.5 FA group: 68.4 ± 3.6 Vitamin B_12_ group: 69.5 ± 2.9 Placebo group: 68.5 ± 3.9	FA + vitamin B_12_ group:39(65.0) FA group:38(63.3) vitamin B_12_ group: 39(65.0) Placebo group: 38(63.3)	400 μg FA and 25 μg vitamin B_12_	6 months	1. Global cognition: FSIQ 2. Memory: digit span 3. Visuospatial skills: block design 4. Attention: digit span 5. Processing speed: digit symbol	FA (ng/mL): FA + vitamin B_12_ group: 7.71 ± 0.26 FA group: 7.71 ± 1.17 Vitamin B_12_ group: 7.55 ± 0.13 Placebo group: 7.61 ± 0.60 Vitamin B12 (pg/mL): FA + vitamin B_12_ group: 401.02 ± 4.49 FA group: 398.32 ± 5.59 vitamin B_12_ group: 403.65 ± 9.97 Placebo group: 405.77 ± 5.15	MMSE: FA + vitamin B_12_ group: 22.07 ± 1.33 FA group: 21.60 ± 1.02 vitamin B_12_ group: 22.13 ± 1.21 Placebo group: 21.95 ± 1.15
He et al. [34]	China	Expert consensus on prevention and treatment of cognitive dysfunction in China [39]	30/26	73.9 ± 7.0/72.1 ± 8.0	17(56.7)/15(57.7)	10.6 mg lycopene and 7.3 mg vitamin B	6 months	Global cognition: BCAT	NA	MMSE: 26.40 ± 3.59/27.62 ± 2.37
Fan et al. [33]	China	Petersen criteria 2004 [23]	40/40	65.5 ± 2.9/66.4 ± 1.9	20(50.0)/22(55.0)	400 μg FA	6 months	Global cognition: MMSE	NA	MMSE: 25.12 ± 1.10/25.03 ± 1.13
Zhang et al. [32]	China	Petersen criteria 2004 [23]	120/120	74.5 ± 2.7/74.6 ± 3.3	77(64.2)/79(65.8)	2 g DHA	12 months	1. Global cognition: FSIQ 2. Memory: digit span 3. Visuospatial skills: block design 4. Attention: digit span 5. Processing speed: digit symbol	DHA mg/d: 103.5 ± 53.6/104.7 ± 49.4	FSIQ: 104.59 ± 6.44/107.51 ± 11.39
Bo et al. [26]	China	Petersen criteria 2004 [23]	44/42	71.8 ± 5.7/70.5 ± 6.8	18(40.9)/17(40.5)	480 mg DHA and 720 mg EPA	6 months	Global cognition: BCAT	DHA (%): 1.55 ± 0.39/1.62 ± 0.43	MMSE: 25.11 ± 1.66/25.62 ± 1.68
Naeini et al. [29]	Iran	An expert psychologist evaluated the subjects to find those with MCI on the basis of attaining scores of 21–26 in the MMSE	127/129	66.5 ± 0.4/66.3 ± 0.4	64(50.4)/72(55.8)	300 mg vitamin E and 400 mg vitamin C	12 months	Global cognition: MMSE	Vitamin E (mg): 12.0 ± 0.9/11.6 ± 0.8 Vitamin C (mg): 90.7 ± 6.4/98.6 ± 5.8	MMSE: *P* = 0.88
Lee et al. [35]	Malaysia	(1) Memory complaints (2) Preserved global cognitive function (3) Intact ADL and IADL (4) Does not meet the criteria for dementia not demented (5) Not depressed (6) Abnormal cognitive impairments for age	17/18	66.4 ± 5.1/63.5 ± 3.0	14(82.4)/13(72.2)	1.3 g DHA and 0.45 mg EPA	12 months	1. Global cognition: MMSE 2. Memory: RAVLT 3. Executive function: CDT 4. Attention: digit span 5. Processing speed: digit symbol substitution test 6. Visuospatial skills: block design	DHA (%): 2.09 ± 1.38/2.02 ± 1.31	MMSE (95% CI): 26.4(25.096–27.658)/26.4(25.127–27.605)
de Jager et al. [27]	United Kingdom	Petersen criteria 2004 [23]	110/113	76.8 ± 5.1/76.7 ± 4.8	70(63.6)/73(64.6)	0.8 mg FA, 0.5 mg vitamin B_12_, and 20 mg vitamin B_6_	24 months	1. Global cognition: MMSE 2. Memory: HVLT-R 3. Executive function: CLOX	FA (95% CI): 22.6(20.0–25.5)/23.0(20.4–26.0) vitamin B_12_ (95% CI)_:_ 332(310–356)/324(303–347)	TICS-M: 24.9 ± 2.8/24.9 ± 2.8

*ADL* activities of daily living, *BCAT* basic cognitive aptitude tests, *CDR-SOB* Clinical Dementia Rating Scale Sum of Boxes, *CDT* clock drawing test, *CG* Control Group, *CLOX* Executive Clock Drawing Task, *DHA* docosahexaenoic acid, *EPA* eicosapentaenoic acid, *FA* Folic Acid, *FSIQ* full-scale intelligence quotient, *GLA* gamma linoleic acid, *HVLT-R* Hopkins Verbal Learning Test-revised, *IADL* instrumental activities of daily living, *IG* Intervention Group, *LA* linoleic acid, *MCI* Mild Cognitive Impairment, *MMSE* Mini-Mental State Examination, *N* Number, *NA* Not Available, *RAVLT* Rey Auditory Verbal Learning Test, *RBANS* the Repeatable Battery for the Assessment of Neuropsychological Status, *%/TFA* total % fatty acid, *TICS-M* Telephone Interview for Cognitive Status-modified, *TMT-A* Trail Making Test A, *TMT-B* Trail Making Test B

The treatments used in the included studies were oral nutrients, including single vitamin B (FA, *n* = 2), multiple B vitamins (combination of FA and vitamin B12, *n* = 2; the combination of FA, vitamin B12, and vitamin B6, *n* = 2), single vitamin D (*n* = 1), multiple vitamins (vitamin C and E compound, *n* = 1; vitamin E and vitamin B compound, *n* = 1), single *n* − 3 PUFA (DHA, *n* = 1), multiple *n* − 3 PUFAs (DHA combined with EPA, *n* = 3), and multiple nutrients of different categories, that is, vitamins combined with PUFAs (FA and DHA compound, *n* = 2; combination of vitamin A, vitamin E, DHA, EPA, gamma linoleic acid [GLA], and linoleic acid [LA], *n* = 1). The treatment duration ranged from 6 to 24 months.

### Risk of bias

**Fig. 2:**
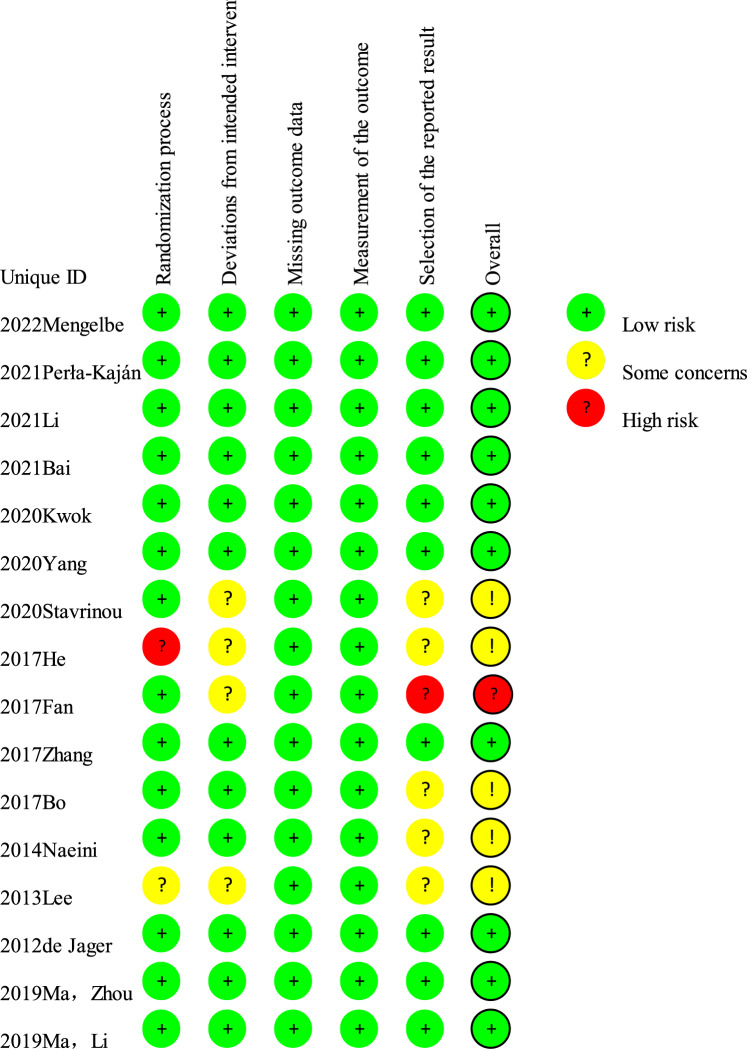
Risk of bias in the included studies

Regarding the overall quality of the included studies, 31.25% had some concerns regarding the risk of bias, 62.5% had a low risk of bias, and 6.25% had a high risk of bias (Fig. 2). One study [33] had a high risk of bias in the selection of the reported result domain, eight studies [19–21, 27, 28, 30–32] had a low risk of bias, and five studies [26, 29, 34–36] had some concerns about the risk of bias, mainly due to deviations from the intended interventions domain and selection of the reported result domain.

### Analysis of primary outcome: global cognitive function

**Fig. 3:**
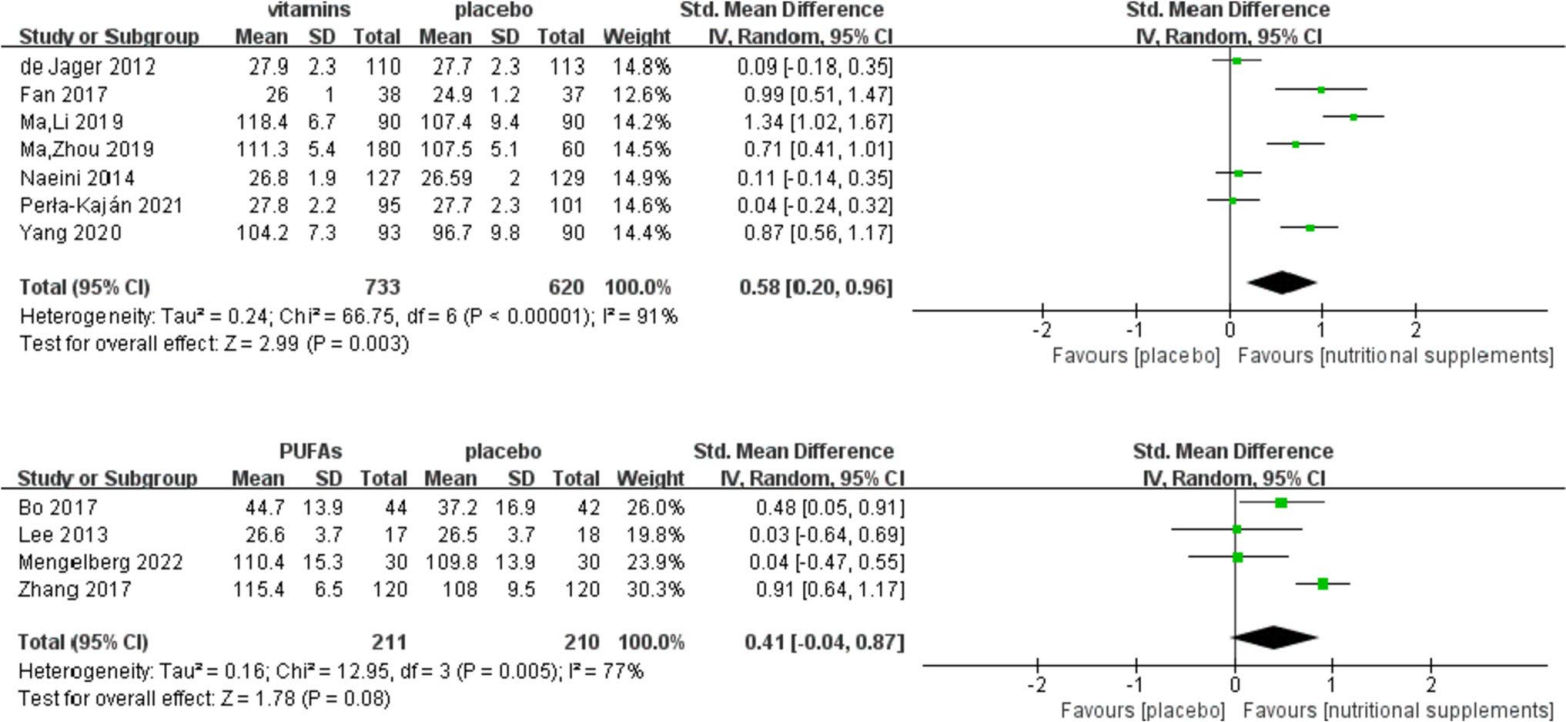
Effect of vitamins and PUFAs on global cognitive function

Sixteen studies [19–21, 26–38] assessed the effects of vitamins and/or PUFAs on global cognitive function in patients with MCI. Of these, five used the MMSE, two used the BCAT, one used the CDR-SOB, one used the RBANS, and seven used the FSIQ to measure global cognitive function. Two studies [34, 37] could not be included in this meta-analysis, as the original data were unavailable. Vitamins might have a beneficial effect on global cognitive function compared with the control interventions (*Z* = 2.99, SMD = 0.58, 95% CI = [0.20, 0.96], *P* = 0.003, Fig. 3).

**Fig. 4:**
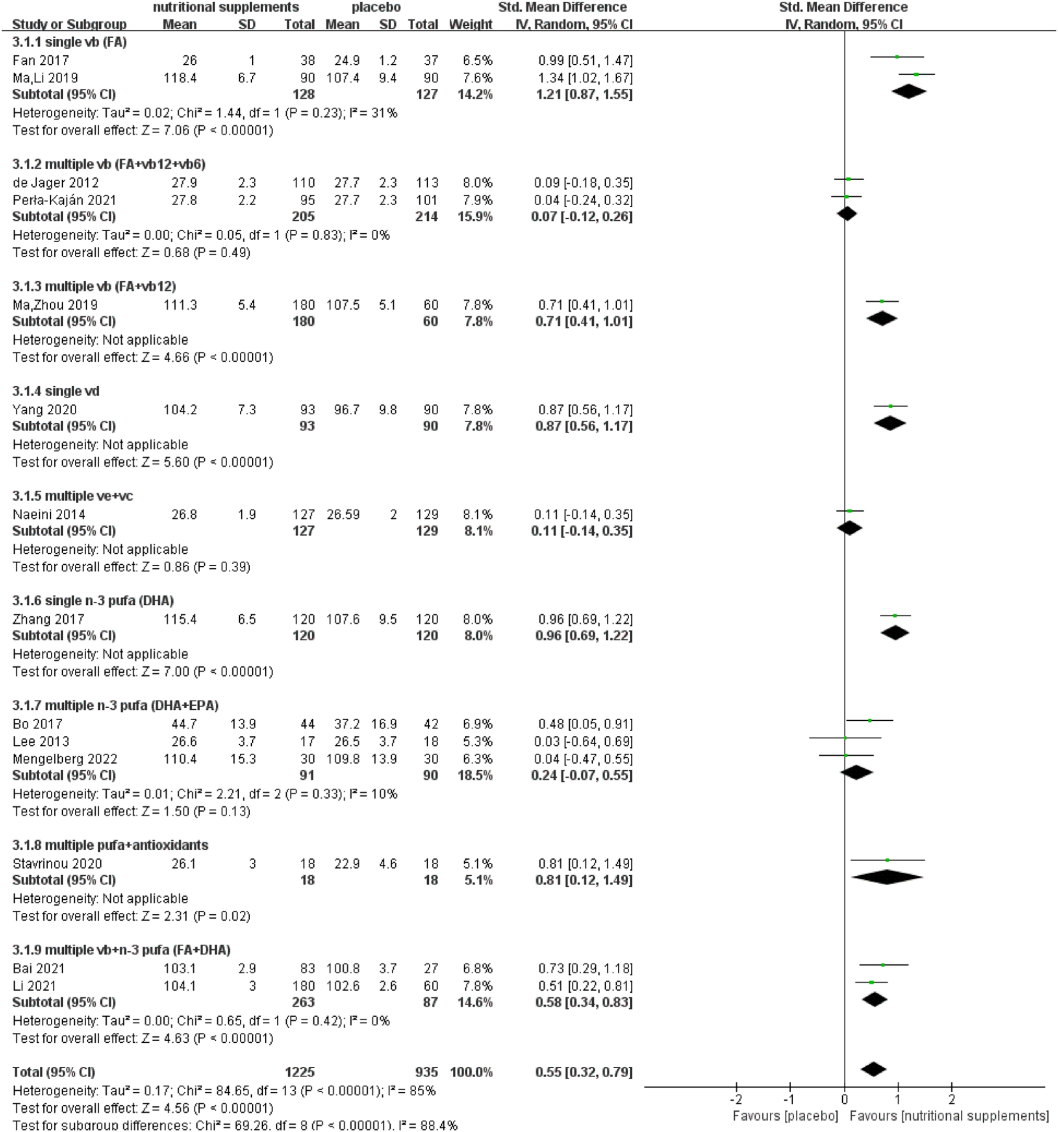
Subgroup analysis of global cognitive function according to nutrient type

We performed a subgroup analysis of different types of vitamins and PUFAs to determine the comparative effects of single and multiple nutrients (Fig. 4).

#### Effect of single and multiple vitamins on global cognitive function

FA [28, 33] exerted a statistically significant effect on global cognitive function (*Z* = 7.06, SMD = 1.21, 95% CI = [0.87, 1.55], *I*^2^ = 31%, *P* < 0.001). Multiple B vitamins (FA combined with vitamin B12) [19] might have a beneficial effect on global cognition (*Z* = 4.66, SMD = 0.71, 95% CI = [0.41, 1.01], *P* < 0.001). However, multiple vitamin B (complex supplements of FA, vitamin B6, and vitamin B12) [27, 30] exerted no statistically significant effect on global cognitive function (Z = 0.68, SMD = 0.07, 95% CI = [− 0.12, 0.26], *I*^2^ = 0%, *P* = 0.49). Subgroup analysis showed that single vitamin B (FA) (SMD = 1.21, 95% CI = [0.87, 1.55]) might have a greater effect on global cognitive function than multiple B vitamins (FA combined with vitamin B12) (SMD = 0.71, 95% CI = [0.41, 1.01]). Only one included study [31] implemented a vitamin D intervention and showed improvement in global cognitive function (*Z* = 5.60, SMD = 0.87, 95% CI = [0.56, 1.17], *P* < 0.001). One study [29] that implemented a combined vitamin C and E intervention did not show any improvement (*Z* = 0.86, SMD = 0.11, 95% CI = [− 0.14, 0.35], *P* = 0.39).

#### Effect of single and multiple n-3 PUFAs on global cognitive function

One study [32] that assessed a single DHA intervention showed improvement in global cognitive function (*Z* = 7.00, SMD = 0.96, 95% CI = [0.69, 1.22], *P* < 0.001). EPA combined with DHA intervention was implemented in three studies [26, 35, 38], and the meta-analysis showed no statistically significant effect on global cognitive function (*Z* = 1.50, SMD = 0.24, 95% CI = [− 0.07, 0.55], *I*^2^ = 10%, *P* = 0.13).

#### Effect of vitamins combined with *n *− 3 PUFAs on global cognitive function

Two studies [20, 21] that used a combined FA and DHA intervention demonstrated significant benefits in global cognitive function (*Z* = 4.63, SMD = 0.58, 95% CI = [0.34, 0.83], *I*^2^ = 0%, *P* < 0.001). Subgroup analysis showed that single FA (SMD = 1.21, 95% CI = [0.87, 1.55]) and single DHA (SMD = 0.96, 95% CI = [0.69, 1.22]) exerted a greater beneficial effect than multiple nutrients of FA and DHA (SMD = 0.58, 95% CI = [0.34, 0.83]). In addition, one study [36] that used PUFAs and antioxidant vitamin-enriched reagents showed a beneficial effect on global cognitive function (Z = 2.31, SMD = 0.81, 95% CI = [0.12, 1.49], *P* = 0.02).

### Subgroup analyses, meta-regression analyses, and sensitivity analysis

We conducted a subgroup analysis according to the nutrient type to investigate clinical heterogeneity (Fig. 4). The *P* value for subgroup differences indicates the interaction between nutrient type and combined effect size. Because several of the included studies were conducted in China, we performed a post hoc subgroup analysis to examine the results concerning the trial region (China region and other regions, Supplementary Fig. S1). This revealed that the 95% CIs did not overlap, and the subgroup differences were statistically significant, indicating that the combined effect size differed by trial region. Subgroup analyses were conducted to examine the influence of participant characteristics, specifically group size and age. Our analyses did not reveal significant interactions when comparing groups with fewer than 50 participants (*N* < 50, 95% CI [0.22, 0.84]) to those with 50 or more participants (*N* > 50, 95% CI [0.25, 0.84]), as illustrated in Supplementary Fig. S2. Similarly, age did not significantly impact the outcomes, with no notable differences between participants younger than 70 years (age < 70, 95% CI [0.26, 0.92]) and those 70 years or older (age > 70, 95% CI [0.18, 0.89]), as depicted in Supplementary Fig. S3. Additionally, meta-regression analyses were performed to investigate heterogeneity attributable to group size, age, and the duration of the nutritional supplement intervention. These analyses showed no significant heterogeneity for group size (*P* = 0.285) or age categories (*P* = 0.143), as evidenced in Supplementary Figs. S4 and S5, respectively. However, a significant interaction was observed across the different intervention durations of 6, 12, and 24 months (*P* < 0.05, Supplementary Fig. S6), indicating that the length of the intervention may play a crucial role in the observed effects. The results remained unchanged after removing studies with a high risk of bias [33].

### Publication bias

**Fig. 5:**
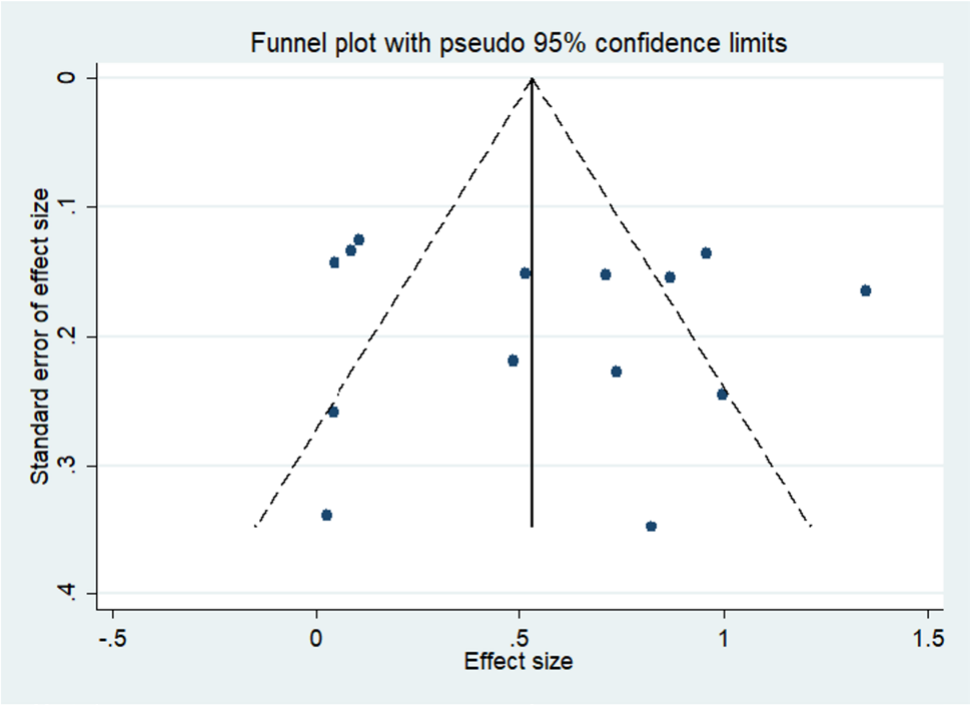
Funnel plot assessing the publication bias of the effects of vitamins and PUFAs on global cognitive function

Figure 5 shows a funnel plot of global cognitive function. Visual inspection of the funnel plots indicated no asymmetry, suggesting that the results of the meta-analysis were not affected by publication bias.

### Analysis of secondary outcomes: memory function

**Fig. 6:**
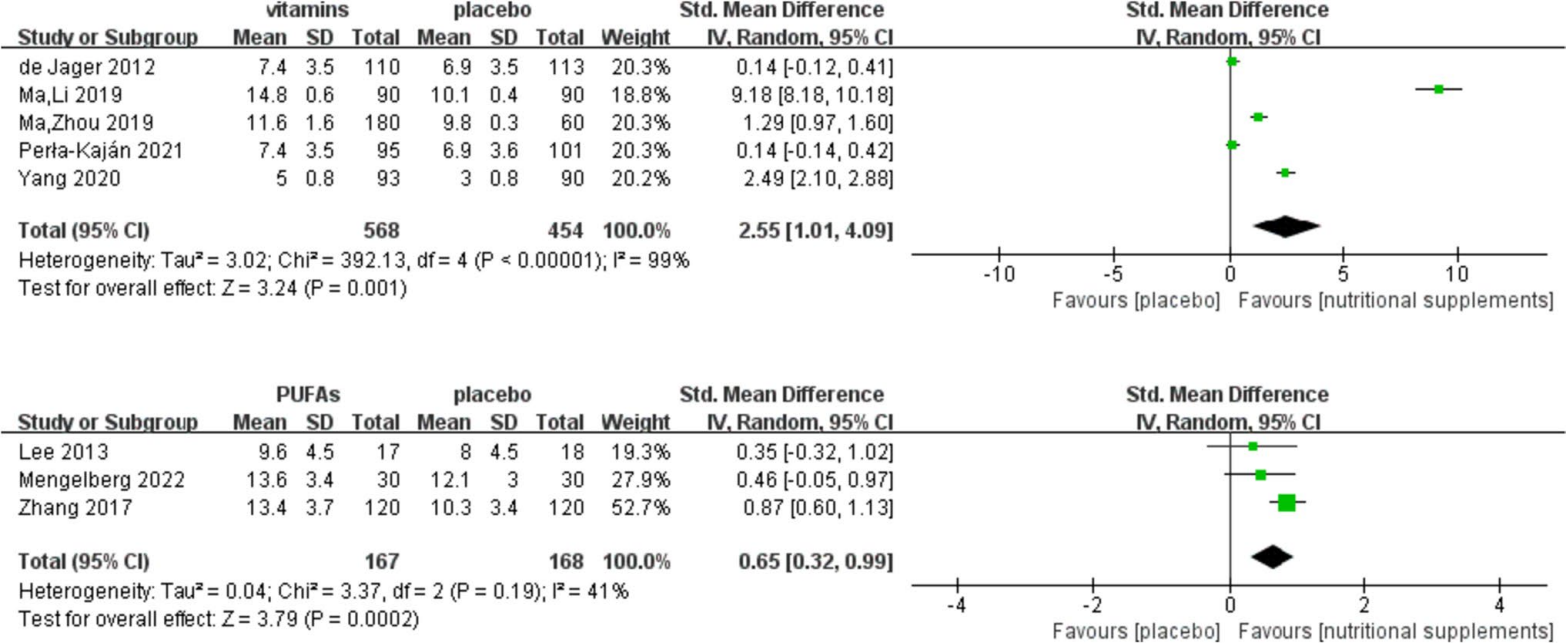
Effect of vitamins and PUFAs on memory

Ten studies [19–21, 27, 28, 30–32, 35, 38] assessed the effects of vitamins and/or PUFAs on memory function in patients with MCI. Meta-analyses showed that the difference in SMDs between the control and intervention groups was statistically significant (vitamins: *Z* = 3.24, SMD = 2.55, 95% CI = [1.01, 4.09], *I*^2^ = 99%, *P* = 0.001; PUFAs: *Z* = 3.79, SMD = 0.65, 95% CI = [0.32, 0.99], *I*^2^ = 41%, *P* < 0.001) (Fig. 6).

**Fig. 7:**
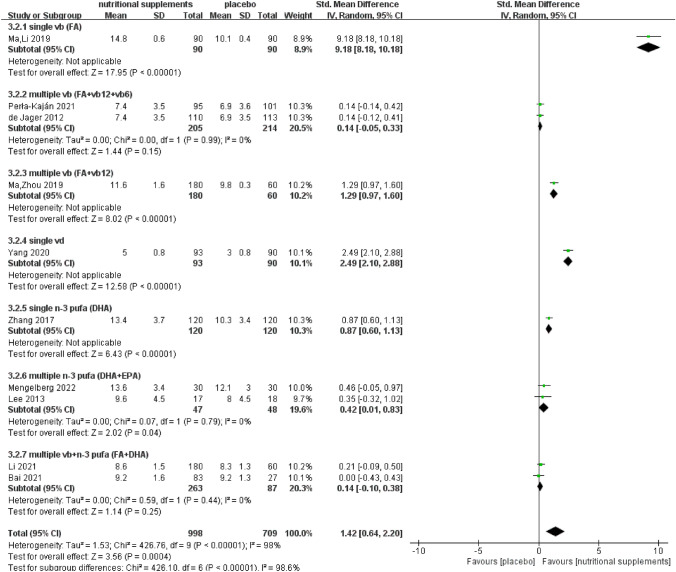
Subgroup analysis of memory function according to nutrient type

A subgroup analysis examining the comparative effects of single and multiple nutrients on memory function is presented in Fig. 7.

#### Effect of single and multiple vitamins on memory function

Only one study [28] implemented a single FA intervention and showed a probable improvement in memory function (*Z* = 17.95, SMD = 9.18, 95% CI = [8.18, 10.18], *P* < 0.001). Multiple B vitamins (FA combined with vitamin B12) [19] might exert a beneficial effect on memory function (*Z* = 8.02, SMD = 1.29, 95% CI = [0.97, 1.60], *P* < 0.001). A meta-analysis of two studies [27, 30] with multiple B vitamin interventions (complex supplements of FA, vitamin B6, and vitamin B12) in patients with MCI did not show a statistically significant effect on memory function (*Z* = 1.44, SMD = 0.14, 95% CI = [− 0.05, 0.33], *I*^2^ = 0%, *P* = 0.15). Subgroup analysis revealed that single FA supplementation (SMD = 9.18, 95% CI = [8.18, 10.18]) might have a greater effect on memory function than multiple vitamin B supplementation (FA and vitamin B12) (SMD = 1.29, 95% CI = [0.97, 1.60]). One study [31] implemented a vitamin D intervention, and the results revealed significant improvement (*Z* = 12.58, SMD = 2.49, 95% CI = [2.10, 2.88], *P* < 0.001).

#### Effect of single and multiple n-3 PUFAs on memory function

One study [32] was performed using a single DHA intervention and revealed a statistically significant effect on memory function (*Z* = 6.43, SMD = 0.87, 95% CI = [0.60, 1.13], *P* < 0.001). Two studies [35] that implemented EPA and DHA interventions showed no statistically significant effect on memory function (*Z* = 2.02, SMD = 0.42, 95% CI = [0.01, 0.83], *I*^2^ = 0%, *P* = 0.04).

#### Effect of vitamins combined with n-3 PUFAs on memory function

Two studies [20, 21] that implemented combined FA and DHA interventions showed no statistically significant effect on memory function (*Z* = 1.14, SMD = 0.14, 95% CI = [− 0.10, 0.38], *I*^2^ = 0%, *P* = 0.25).

### Sensitivity analyses

Subgroup analysis according to the type of nutrient (Fig. 7) revealed an overlap in the 95% CIs; however, the effect sizes differed (*P* < 0.05). This indicated that an interaction existed between the type of nutrient and its effect on memory function, that is, the nutrient type was the main source of heterogeneity.

### Analysis of secondary outcomes: attention

**Fig. 8:**
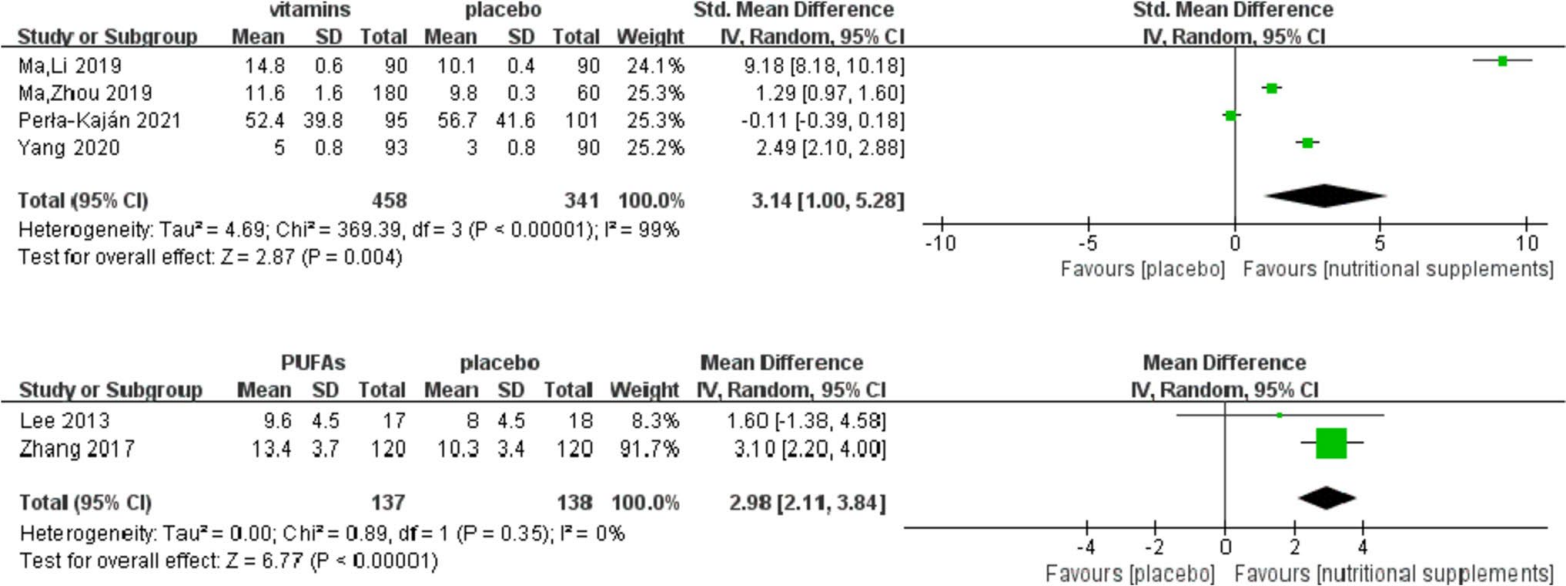
Effect of vitamins and PUFAs on attention

Different instruments were used to measure attention in nine studies [19–21, 28, 30–32, 35, 36], and meta-analyses of these studies revealed that vitamins and/or PUFAs significantly affected attention in patients with MCI (vitamins: *Z* = 2.87, SMD = 3.14, 95% CI = [1.00, 5.28], *I*^2^ = 99%, *P* = 0.004; PUFAs: *Z* = 6.77, SMD = 2.98, 95% CI = [2.11, 3.84], *I*^2^ = 0%, *P* < 0.001), as shown in Fig. 8.

**Fig. 9:**
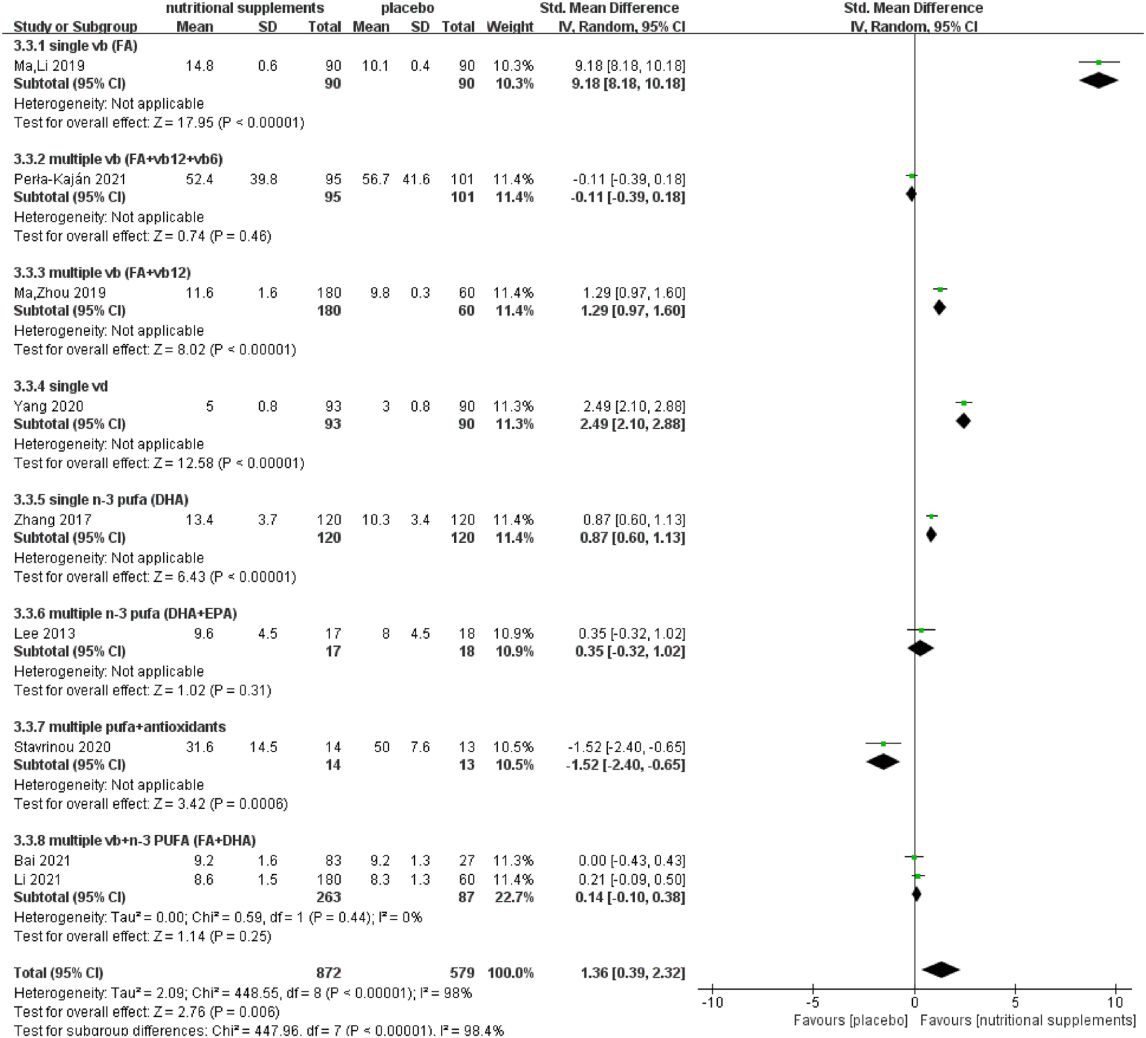
Subgroup analysis of attention according to nutrient type

A subgroup analysis assessing the comparative effects of single and multiple nutrients on attention is presented in Fig. 9.

#### Effect of single and multiple vitamins on attention

A single FA intervention was only administered to patients with MCI in one study [28], and meta-analysis revealed a statistically significant effect on attention (*Z* = 17.95, SMD = 9.18, 95% CI = [8.18, 10.18], *I*^2^ = 22%, *P* < 0.001). Multiple vitamin B (FA combined with vitamin B12) [19] exerted a statistically significant effect on attention (*Z* = 8.02, SMD = 1.29, 95% CI = [0.97, 1.60], *P* < 0.001). A multiple vitamin B intervention (complex supplements of FA, vitamin B6, and vitamin B12) was performed in another study [30], which revealed no statistically significant effect on attention (*Z* = 0.74, SMD = − 0.11, 95% CI = [− 0.39, 0.18], *P* = 0.46). Subgroup analysis showed that single FA intake (SMD = 9.18, 95% CI = [8.18, 10.18]) might have a greater effect on attention than multiple vitamin B intake (SMD = 1.29, 95% CI = [0.97, 1.60]). One study that implemented a vitamin D intervention [31] showed a statistically significant effect on attention (*Z* = 12.58, SMD = 2.49, 95% CI = [2.10, 2.88], *P* < 0.001).

#### Effect of single and multiple n-3 PUFAs on attention

One study that performed a single DHA intervention [32] showed a statistically significant effect on attention (*Z* = 6.43, SMD = 0.87, 95% CI = [0.60, 1.13], *P* < 0.001). Another study that implemented EPA and DHA interventions [35] showed no statistically significant effect on attention (*Z* = 1.02, SMD = 0.35, 95% CI = [− 0.32, 1.02], *P* = 0.31).

#### Effect of vitamin combined with *n *− 3 PUFAs on attention

Two studies that implemented a combination of FA and DHA interventions [20, 21] showed no improvement in attention (*Z* = 1.14, SMD = 0.14, 95% CI = [− 0.10, 0.38], *I*^2^ = 0%, *P* = 0.25). A study that used a combination of EPA, DHA, and antioxidant vitamins [36] showed no statistically significant effect on attention (*Z* = 3.42, SMD = − 1.52, 95% CI = [− 2.40, − 0.65], *P* < 0.001).

### Sensitivity analyses

Subgroup analysis according to the type of nutrient (Fig. 9) revealed an overlap in the 95% CIs. However, the effect sizes differed (*P* < 0.05), indicating an interaction between the type of nutrient and its effect on attention, that is, the nutrient type was the main source of heterogeneity.

### Analysis of secondary outcomes: visuospatial skills

Different instruments were used in seven studies [19–21, 28, 31, 32, 35] to measure visuospatial skills, and meta-analyses of these studies revealed that vitamins and/or PUFAs did not significantly affect visuospatial skills (vitamins: *Z* = 1.43, SMD = 0.97, 95% CI = [-0.36, 2.29], *I*^2^ = 85%, *P* = 0.15; PUFAs: *Z* = 1.18, SMD = 0.63, 95% CI = [-0.41, 1.67], *I*^2^ = 4%, *P* = 0.24, Supplementary Fig. S7), with high heterogeneity.

### Analysis of secondary outcomes: executive function

Different instruments were used in three studies [27, 35, 36] to measure executive function, and a meta-analysis of these studies revealed no statistically significant effect of vitamins and/or PUFAs on executive function (vitamins: *Z* = 1.49, SMD = 0.40, 95% CI = [− 0.13, 0.93], *P* = 0.14; PUFAs: *Z* = 0.84, SMD = − 0.17, 95% CI = [− 0.58, 0.23], *I*^2^ = 0%, *P* = 0.40, Supplementary Fig. S8), with high heterogeneity.

### Analysis of secondary outcomes: processing speed

Different instruments were used in six studies [19, 30–32, 35, 36] to measure processing speed, and meta-analyses of these studies revealed that vitamins and/or PUFAs did not affect processing speed significantly (vitamins: *Z* = 0.21, SMD = 0.02, 95% CI = [− 0.13, 0.16], *I*^2^ = 0%, *P* = 0.83; PUFAs: *Z* = 2.43, SMD = 0.80, 95% CI = [0.15, 1.44], *I*^2^ = 0%, *P* = 0.02, Supplementary Fig. S9), with slight heterogeneity.

## Discussion

### Main findings

The effect of vitamins and PUFAs in patients with MCI was examined, and the effect sizes of single and multiple nutrients were compared in the present study. Sixteen studies that enrolled 2528 patients with MCI were included. Our results suggest that FA, vitamin B6, vitamin B12, and vitamin D might positively affect global cognitive function, memory function, and attention, but not visuospatial skills, executive function, and processing speed, in patients with MCI. EPA and DHA may improve memory function and attention in patients with MCI. Single FA might have a greater effect on global cognitive function, memory function, and attention than a vitamin B combination (FA and vitamin B12) or the combination of FA and DHA.

### Effects of vitamins on clinical outcomes

The effects of vitamin supplementation on cognition have become a topic of increasing interest. We analyzed the effects of single and multiple vitamin B supplements on global cognitive function and various cognitive domains in patients with MCI. Our meta-analysis indicates potential cognitive improvements associated with FA, vitamin B6, and vitamin B12, particularly in global cognitive function, memory, and attention. Notably, FA alone might offer more cognitive benefits than a combination of multiple B vitamins. The improvement in cognitive function was associated with the ability of vitamin B to reduce homocysteine concentration [40, 41]. Homocysteine is a risk factor for cognitive decline and the progression to dementia [42]. In addition, FA and vitamin B12 levels interact with each other due to factors such as vitamin metabolism, implying that the effect of one vitamin B on cognitive function may be altered by the blood concentration of another [19]. Variations in study design, sample size, and participant demographics have led to conflicting conclusions in meta-analyses evaluating the effects of vitamin B on cognition. For example, contrasting evidence on vitamin B's benefit for memory function emerged from meta-analyses with vastly different sample sizes and participant profiles [16, 18]. Given the results of this meta-analysis, clinical staff should consider vitamin B interactions when implementing nutritional supplementation intervention programs in patients with MCI and prioritize single FA interventions. However, additional studies on vitamin B interventions of different doses and durations in older adults with MCI are needed to confirm this interpretation, as the data supporting this interpretation are insufficient.

Vitamin D is also related to cognition. Our meta-analysis included one study that examined the effect of vitamin D on cognition [31]. The results demonstrated that vitamin D positively affected global cognitive function, memory, and attention in patients with MCI. This is similar to the results of a previous meta-analysis based on observational studies [43] that found a correlation between low vitamin D levels and cognitive decline. However, this previous meta-analysis also included intervention studies and did not find a positive effect of vitamin D on cognitive function. It included three interventional studies of vitamin D with a maximum duration of 6 weeks. In comparison, our meta-analysis included studies that were conducted over 12 months. In addition, the previous meta-analysis included older adults without a dementia diagnosis, more than MCI, which did not fit our purpose. The results of this review still need to be confirmed, however, due to the limited number of original studies. We also, unfortunately, failed to explore the effect sizes of single versus combined vitamin D owing to insufficient original studies and did not provide a guide for future research directions. This review also included one study [29] that implemented oral vitamin C combined with vitamin E interventions, which did not significantly affect cognitive function. The findings were similar to those of a meta-analysis conducted in 2017 [44]; however, including studies with small samples meant that the conclusions were not robust.

## Effects of *n *− 3 PUFAs on clinical outcomes

Three studies [26, 35, 38] implementing combined DHA and EPA intervention and one [32] implementing DHA monotherapy were included in this meta-analysis. Meta-analyses showed that DHA alone may improve global cognitive function, memory function, and attention, whereas DHA and EPA intervention did not positively affect cognitive function. A study has shown that *n *− 3 PUFAs improve hippocampal structure, promote neurodevelopment, and enhance brain function [45]. The anti-inflammatory properties of *n *− 3 PUFAs, such as DHA and EPA, can reduce inflammation-related cognitive decline by inhibiting inflammatory responses in microglia [45]. However, the exact nature of the interaction between DHA and EPA remains unclear. Most existing meta-analyses have aimed to investigate the association of multiple *n *− 3 PUFAs or single DHA with cognition, but no comparative analysis of single and multiple *n *− 3 PUFAs has been performed [46–48]. Our findings underscore the need for healthcare providers to discern the distinct impacts of single versus combined *n *− 3 PUFA interventions when formulating nutrition therapy plans for older adults with MCI. Nevertheless, after exploring the original study, we found that Lee et al. [35] included only 35 patients with MCI. Because of the small sample size, the results must be interpreted with caution. Large-scale studies are needed to solidify this evidence.

### Effects of the combination of vitamins and *n *− 3 PUFAs on clinical outcomes

This meta-analysis included three studies that implemented combined vitamins and PUFAs (two studies of FA combined with DHA [20, 21] and one study on vitamin A, vitamin E, DHA, EPA, GLA, and LA [36]). These results suggest that the combined vitamin and PUFA intervention positively affected global cognitive function. However, contrary to the original trials, our subgroup analysis of nutritional types revealed that FA and DHA in combination were less effective than either isolated compound. This may be related to different blood concentrations of n-3 PUFAs influencing the effect of FA on cognitive function [49, 50]. Healthcare professionals should weigh both the nutritional status of patients and economic factors when choosing nutritional supplementation therapies. However, these recommendations are tentative, grounded in limited trials, and should be applied cautiously until further evidence-based research is available.

### Implications

More RCTs, rather than cross-sectional or case–control studies, are needed in the future to determine the effect of vitamins and PUFAs and the comparative effect between single and multiple nutrients on cognitive function. Furthermore, the limited statistical power of small-scale studies necessitates larger trials to verify the significance of our findings. The variety of cognitive assessment tools used, such as the MMSE or MoCA, complicates the interpretation of clinical relevance. Study designs that focus on consistent baseline assessments and intervention durations are critical for establishing robust conclusions.

### Strengths and limitations

The advantage of our study is that we are the first to our knowledge to systematically compare the effects of multiple and single nutrients on cognitive function. Moreover, we strictly controlled the screening criteria for the population with MCI, thereby increasing the reliability of the conclusions. Our study also has several shortcomings that necessitate a cautious interpretation of the findings. First, because our systematic review and meta-analysis included only English and Chinese trials, it failed to include sufficient original studies. Some results may be missing. In addition, owing to the use of different assessment tools for cognitive function and domains, differences between the original studies may be difficult to detect, despite the reduction in bias in this meta-analysis using SMD effect models. Moreover, as most studies were conducted in China, we performed a post hoc subgroup analysis, which indicated that heterogeneity was caused by different regions of origin in each study. Therefore, caution should be exercised when generalizing our findings to other regions. Finally, the baseline blood concentrations of nutrients, the baseline cognition of participants, the doses of nutrients, and intervention duration among the eligible studies were inconsistent, causing great heterogeneity that could not be addressed using sensitivity analyses. Therefore, these differences should be noted when interpreting the results.

## Conclusion

In this meta-analysis, we found that FA, vitamin B6, vitamin B12, and vitamin D might positively affect global cognitive function, memory function, and attention, but not visuospatial skills, executive function, and processing speed, in patients with MCI. EPA and DHA may improve memory function and attention in patients with MCI. FA may be more effective than vitamin B complex (FA, vitamin B12, and/or vitamin B6) in terms of global cognitive function, memory function, and attention. Future RCTs that are conducted on MCI patients and control for supplementation type, dose, and duration of the intervention are needed to confirm this conclusion and explore the detailed application of vitamins and PUFAs in clinical practice.

### Supplementary Information

Below is the link to the electronic supplementary material.

Supplementary file1 (DOCX 234 KB)

## Data Availability

Not applicable.
