# Supplementary material for: Effects of vitamins and polyunsaturated fatty acids on cognitive function in older adults with mild cognitive impairment: a meta-analysis of randomized controlled trials
Source: Eur J Nutr. 2024 Feb 1;63(4):1003–22. doi: 10.1007/s00394-024-03324-y (PMC11139751; doi:10.1007/s00394-024-03324-y)
Supplement: Supplementary file 1 — Supplementary file1 (DOCX 234 KB) [file 394_2024_3324_MOESM1_ESM.docx]

**Effects of Vitamins and Polyunsaturated Fatty Acids on Cognitive Function in Older Adults with Mild Cognitive Impairment: A Meta-Analysis of Randomized Controlled Trials**

Jing Chang^1†^, Minhui Liu ^2†^, Chang Liu^1^, Shiyu Zhou^1^, Yuchen Jiao^1^, Hongyu Sun^3*^, Yan Ji^1*^

^1^School of Nursing, Nanjing Medical University, 101 Longmian Avenue, Jiangning District, Nanjing, Jiangsu, 211166, China.

^2^Xiangya School of Nursing, Central South University, 172 Tongzipo Road of Yuelu District, Changsha, 410013, China.

^3^School of Nursing, Peking University, 38 College Road, Haidian District, Beijing, 100191, China.

^†^Both authors contributed equally to this work.

^*^Corresponding authors. Emails: sunhongyu@bjmu.edu.cn; yanji@njmu.edu.cn;

Contributing authors: cjzmfs@163.com; mliu62@jhu.edu; walnut1997@163.com; zsy20000927@163.com; jyc12342021@163.com

**Supplemental Table S1. Search Strategy.**

**Cochrane**

| Search | Query | Results |
| --- | --- | --- |
| #1 | MeSH descriptor: [Cognitive Dysfunction] explode all trees | 2993 |
| #2 | ("mild cognitive impairment" or "cognitive dysfunction" or "cognitive defect" or "cognitive decline" or "cognitive impairment" or "mild neurocognitive disorder" or "mental deterioration" or "age associated cognitive decline" or "pre-dementia"):ti,ab,kw (Word variations have been searched) | 19042 |
| #3 | MeSH descriptor: [Dietary Supplements] explode all trees | 16970 |
| #4 | MeSH descriptor: [Nutrition Therapy] explode all trees | 12092 |
| #5 | ("dietary supplement" or "nutrition therapy" or "nutritional supplement" or "nutrition" or "nutrient"):ti,ab,kw (Word variations have been searched) | 40037 |
| #6 | MeSH descriptor: [Vitamin A] explode all trees | 2357 |
| #7 | MeSH descriptor: [Vitamin B Complex] explode all trees | 1156 |
| #8 | MeSH descriptor: [Folic Acid] explode all trees | 4218 |
| #9 | MeSH descriptor: [Ascorbic Acid] explode all trees | 2579 |
| #10 | MeSH descriptor: [Antioxidants] explode all trees | 5902 |
| #11 | MeSH descriptor: [Vitamin D] explode all trees | 6928 |
| #12 | MeSH descriptor: [Vitamin E] explode all trees | 2867 |
| #13 | MeSH descriptor: [Vitamin K] explode all trees | 771 |
| #14 | MeSH descriptor: [Fatty Acids] explode all trees | 25967 |
| #15 | MeSH descriptor: [Fatty Acids, Omega-3] explode all trees | 3804 |
| #16 | MeSH descriptor: [Fatty Acids, Omega-6] explode all trees | 864 |
| #17 | MeSH descriptor: [Eicosapentaenoic Acid] explode all trees | 1233 |
| #18 | MeSH descriptor: [Arachidonic Acid] explode all trees | 1958 |
| #19 | MeSH descriptor: [Docosahexaenoic Acids] explode all trees | 1425 |
| #20 | ("vitamin A" or "VA" or "vitamin B" or "VB" or "folic acid" or "FA" or "Vitamin B9" or "B9" or "Vitamin B6" or "B6" or "Vitamin B12" or "B12" or "vitamin C" or "VC" or "antioxidant*" or "vitamin D" or "VD" or "vitamin E" or "VE" or "vitamin K" or "VK" or "fatty acid" or "PUFA" or "omega 3 fatty acid" or " docosahexaenoic acid" or "DHA" or "α-Linolenic acid" or "ALA" or "eicosapentaenoic acid" or "EPA" or "omega 6 fatty acid" or "arachidonic acid" or "AA" or "linoleic acid" or "LA" or "γ- Linolenic acid" or "GLA"):ti,ab,kw (Word variations have been searched)("vitamin B" or "antioxidant* " or "vitamin D" or "fatty acid*" or "omega 3 fatty acid*"):ti,ab,kw (Word variations have been searched) | 91069 |
| #21 | MeSH descriptor: [Randomized Controlled Trial] explode all trees | 25732 |
| #22 | MeSH descriptor: [Controlled Clinical Trial] explode all trees | 38477 |
| #23 | ("randomized controlled trial" or "randomized clinical trial" or "controlled clinical trial" or "randomized"):ti,ab,kw (Word variations have been searched) | 1126948 |
| #24 | #1 or #2 | 19057 |
| #25 | #3 or #4 or #5 or #6 or #7 or #8 or #9 or #10 or #11 or #12 or #13 or #14 or #15 or #16 or #17 or #18 or #19 or #20 | 164221 |
| #26 | #21 or #22 or #23 | 1126948 |
| #27 | #24 and #25 and #26 | 1306 |

**PubMed**

| Search | Query | Results |
| --- | --- | --- |
| #1 | "Cognitive Dysfunction"[MeSH Terms] | 36053 |
| #2 | "cognitive dysfunction*"[Title/Abstract] OR "mild cognitive impairment*"[Title/Abstract] OR "cognitive defect"[Title/Abstract] OR "cognitive declin*"[Title/Abstract] OR "cognitive impairment*"[Title/Abstract] OR "mild neurocognitive disorder*"[Title/Abstract] OR "mental deterioration*"[Title/Abstract] OR "age associated cognitive decline"[Title/Abstract] OR "pre-dementia"[Title/Abstract] | 129182 |
| #3 | #1 OR #2 | 135144 |
| #4 | "Dietary Supplements"[MeSH Terms] OR "Nutrition Therapy"[MeSH Terms] | 210837 |
| #5 | "dietary supplement*"[Title/Abstract] OR "nutrition therapy"[Title/Abstract] OR "nutritional supplement*"[Title/Abstract] OR "nutrition*"[Title/Abstract] OR "nutrient"[Title/Abstract] | 495345 |
| #6 | "Diet, Food, and Nutrition"[Mesh] OR "Vitamin A"[Mesh] OR "Vitamin B Complex"[Mesh] OR "Vitamin B 6"[Mesh] OR "Vitamin B 12"[Mesh] OR "[Folic Acid](https://www.ncbi.nlm.nih.gov/mesh/68005492)"[Mesh] OR "Antioxidants"[Mesh] OR "[Ascorbic Acid](https://www.ncbi.nlm.nih.gov/mesh/68001205)"[Mesh] OR "Vitamin D"[Mesh] OR "Vitamin E"[Mesh] OR "Vitamin K"[Mesh] OR "Fatty Acids"[Mesh] OR "Fatty Acids, Omega-3"[Mesh] OR "Docosahexaenoic Acids"[Mesh] OR "alpha-Linolenic Acid "[Mesh] OR "[Eicosapentaenoic Acid](https://www.ncbi.nlm.nih.gov/mesh/68015118)"[Mesh] OR "Fatty Acids, Omega-6"[Mesh] OR "[Arachidonic Acid](https://www.ncbi.nlm.nih.gov/mesh/68016718)"[Mesh] OR "[Linoleic Acid](https://www.ncbi.nlm.nih.gov/mesh/68019787)"[Mesh] OR "[gamma-Linolenic Acid](https://www.ncbi.nlm.nih.gov/mesh/68017965)"[Mesh] | 1990912 |
| #7 | "vitamin A"[Title/Abstract] OR "VA"[Title/Abstract] OR "vitamin B"[Title/Abstract] OR "VB"[Title/Abstract] OR "folic acid"[Title/Abstract] OR "FA"[Title/Abstract] OR "Vitamin B9"[Title/Abstract] OR "B9"[Title/Abstract] OR "Vitamin B6"[Title/Abstract] OR "B6"[Title/Abstract] OR "Vitamin B12"[Title/Abstract] OR "B12"[Title/Abstract] OR "antioxidant*"[Title/Abstract] OR "vitamin C"[Title/Abstract] OR "VC"[Title/Abstract] OR "vitamin D"[Title/Abstract] OR "VD"[Title/Abstract] OR "vitamin E"[Title/Abstract] OR "VE"[Title/Abstract] OR "vitamin K"[Title/Abstract] OR "VK"[Title/Abstract] OR "fatty acid*"[Title/Abstract] OR "PUFA"[Title/Abstract] OR "omega 3 fatty acid*"[Title/Abstract] OR "docosahexaenoic acid"[Title/Abstract] OR "DHA"[Title/Abstract] OR "α-Linolenic acid"[Title/Abstract] OR "ALA"[Title/Abstract] OR "eicosapentaenoic acid"[Title/Abstract] OR "EPA"[Title/Abstract] OR "omega 6 fatty acid*"[Title/Abstract] OR "arachidonic acid"[Title/Abstract] OR "AA"[Title/Abstract] OR "linoleic acid"[Title/Abstract] OR "LA"[Title/Abstract] OR "γ- Linolenic acid"[Title/Abstract] OR "GLA"[Title/Abstract] | 1080681 |
| #8 | #4 OR #5 OR #6 OR #7 | 2858518 |
| #9 | "Randomized Controlled Trial" [Publication Type] OR "Controlled Clinical Trial" [Publication Type] | 688039 |
| #10 | "randomized controlled trial*"[Title/Abstract] OR "controlled clinical trial*"[Title/Abstract] OR "randomized clinical trial*"[Title/Abstract] OR "randomized"[Title/Abstract] | 678474 |
| #11 | #9 OR #10 | 1034167 |
| #12 | #3 AND # 8 AND #11 | 1076 |

**EMBASE**

| Search | Query | Results |
| --- | --- | --- |
| #1 | 'mild cognitive impairment'/exp OR 'cognitive defect'/exp | 613014 |
| #2 | 'mild cognitive impairment':ab,ti OR 'cognitive defect':ab,ti OR 'cognitive dysfunction':ab,ti OR 'cognitive declin*':ab,ti OR 'mild neurocognitive disorder*':ab,ti OR 'mental deterioration*':ab,ti OR 'age associated cognitive decline':ab,ti OR 'pre-dementia':ab,ti | 103421 |
| #3 | #1 OR #2 | 627636 |
| #4 | 'dietary supplement'/exp OR 'diet supplementation'/exp OR 'nutrition'/exp OR 'nutrition supplement'/exp | 2786562 |
| #5 | 'dietary supplement*':ab,ti OR 'diet supplementation':ab,ti OR 'nutrition':ab,ti OR 'nutrition supplement*':ab,ti OR 'nutrient*':ab,ti | 483159 |
| #6 | 'vitamin B group'/exp OR 'folic acid'/exp OR 'antioxidant'/exp OR 'ascorbic acid'/exp OR 'vitamin d'/exp OR 'vitamin K group'/exp OR 'multivitamin'/exp OR 'fatty acid'/exp OR 'omega 3 fatty acid'/exp OR 'docosahexaenoic acid'/exp OR 'linolenic acid'/exp OR 'icosapentaenoic acid'/exp OR 'omega 6 fatty acid'/exp OR 'arachidonic acid'/exp OR 'linoleic acid'/exp | 1557268 |
| #7 | 'vitamin A':ab,ti OR 'VA':ab,ti OR 'vitamin B':ab,ti OR 'VB':ab,ti OR 'folic acid':ab,ti OR 'FA':ab,ti OR 'vitamin B9':ab,ti OR 'B9':ab,ti OR 'vitamin B6':ab,ti OR 'B6':ab,ti OR 'vitamin B12':ab,ti OR 'B12':ab,ti OR 'antioxidant*':ab,ti OR 'vitamin C':ab,ti OR 'VC':ab,ti OR 'vitamin d':ab,ti OR 'VD':ab,ti OR 'vitamin E':ab,ti OR 'VE':ab,ti OR 'vitamin K':ab,ti OR 'VK':ab,ti OR 'fatty acid*':ab,ti OR 'PUFA':ab,ti OR 'omega 3 fatty acid':ab,ti OR 'docosahexaenoic acid':ab,ti OR 'DHA':ab,ti OR 'α-Linolenic acid':ab,ti OR 'ALA':ab,ti OR 'eicosapentaenoic acid':ab,ti OR 'EPA':ab,ti OR 'omega 6 fatty acid':ab,ti OR 'arachidonic acid':ab,ti OR 'AA':ab,ti OR 'linoleic acid':ab,ti OR 'LA':ab,ti OR 'γ- Linolenic acid':ab,ti OR 'GLA':ab,ti | 1425166 |
| #8 | #4 OR #5 OR #6 OR #7 | 4819496 |
| #9 | 'randomized controlled trial'/exp OR 'controlled clinical trial'/exp | 955680 |
| #10 | 'randomized controlled trial*':ab,ti OR 'controlled clinical trial*':ab,ti OR 'randomized clinical trial*':ab,ti OR 'randomized':ab,ti | 968119 |
| #11 | #9 OR #10 | 1440179 |
| #12 | #3 AND #8 AND #11 | 4396 |

**CINAHL**

| Search | Query | Results |
| --- | --- | --- |
| S1 | (MH "Mild Cognitive Impairment") | 451 |
| S2 | TI ("mild cognitive impairment*" or "cognitive dysfunction*" or "cognitive defect" or "cognitive declin*" or "cognitive impairment*" or "mild neurocognitive disorder*" or "mental deterioration*" or "age associated cognitive decline" or "pre-dementia") OR AB ("mild cognitive impairment*" or "cognitive dysfunction*" or "cognitive defect" or "cognitive declin*" or "cognitive impairment*" or "mild neurocognitive disorder*" or "mental deterioration*" or "age associated cognitive decline" or "pre-dementia") OR SU ("mild cognitive impairment*" or "cognitive dysfunction*" or "cognitive defect" or "cognitive declin*" or "cognitive impairment*" or "mild neurocognitive disorder*" or "mental deterioration*" or "age associated cognitive decline" or "pre-dementia") | 28050 |
| S3 | (MH "Dietary Supplements+") OR (MH "Dietary Supplementation") | 31075 |
| S4 | (MH "Nutrition+") | 105236 |
| S5 | TI ("dietary supplement*" or "nutrition therapy" or "nutritional supplement*" or "nutrition*" or "nutrient") OR AB ("dietary supplement*" or "nutrition therapy" or "nutritional supplement*" or "nutrition*" or "nutrient") OR SU ("dietary supplement*" or "nutrition therapy" or "nutritional supplement*" "nutrition*" or "nutrient") | 10649 |
| S6 | (MH "Vitamin A+") | 3136 |
| S7 | (MH "Vitamin B Complex+") | 12557 |
| S8 | (MH "Folic Acid+") | 6521 |
| S9 | (MH "Antioxidants+") | 15228 |
| S10 | (MH "Ascorbic Acid+") | 3671 |
| S11 | (MH "Vitamin D+") | 13204 |
| S12 | (MH "Vitamin E+") | 3499 |
| S13 | (MH "Vitamin K+") | 1834 |
| S14 | (MH "Fatty Acids+") | 27424 |
| S15 | (MH "Fatty Acids, Omega-3+") | 6996 |
| S16 | (MH "Docosahexaenoic Acids+") | 2126 |
| S17 | (MH "alpha-Linolenic Acid+") | 396 |
| S18 | (MH "Linolenic Acids+") | 707 |
| S19 | (MH "Eicosapentaenoic Acid+") | 1414 |
| S20 | (MH "Fatty Acids, Omega-6+") | 1701 |
| S21 | (MH "Arachidonic Acids+") | 5220 |
| S22 | (MH "Linoleic Acids+") | 974 |
| S23 | (MH "gamma-Linolenic Acid+") | 86 |
| S24 | TI ("vitamin A" or "VA" or "vitamin B" or "VB" or "folic acid" or "FA" or "Vitamin B9" or " B9" or "Vitamin B6" or "B6" or "Vitamin B12" or "B12" or "antioxidant* " or "vitamin C" or "VC" or "vitamin D" or "VD" or "vitamin E" or "VE" or "vitamin K" or "VK" or "fatty acid*" or "PUFA" or "omega 3 fatty acid*" or "docosahexaenoic acid" or "DHA" or "α-Linolenic acid" or "ALA" or "eicosapentaenoic acid" or "EPA" or "omega 6 fatty acid*" or "arachidonic acid" or "AA" or "linoleic acid" or "LA" or "γ- Linolenic acid" or "GLA") OR AB ("vitamin A" or "VA" or "vitamin B" or "VB" or "folic acid" or "FA" or "Vitamin B9" or " B9" or "Vitamin B6" or "B6" or "Vitamin B12" or "B12" or "antioxidant* " or "vitamin C" or "VC" or "vitamin D" or "VD" or "vitamin E" or "VE" or "vitamin K" or "VK" or "fatty acid*" or "PUFA" or "omega 3 fatty acid*" or "docosahexaenoic acid" or "DHA" or "α-Linolenic acid" or "ALA" or "eicosapentaenoic acid" or "EPA" or "omega 6 fatty acid*" or "arachidonic acid" or "AA" or "linoleic acid" or "LA" or "γ- Linolenic acid" or "GLA") OR SU ("vitamin A" or "VA" or "vitamin B" or "VB" or "folic acid" or "FA" or "Vitamin B9" or " B9" or "Vitamin B6" or "B6" or "Vitamin B12" or "B12" or "antioxidant* " or "vitamin C" or "VC" or "vitamin D" or "VD" or "vitamin E" or "VE" or "vitamin K" or "VK" or "fatty acid*" or "PUFA" or "omega 3 fatty acid*" or "docosahexaenoic acid" or "DHA" or "α-Linolenic acid" or "ALA" or "eicosapentaenoic acid" or "EPA" or "omega 6 fatty acid*" or "arachidonic acid" or "AA" or "linoleic acid" or "LA" or "γ- Linolenic acid" or "GLA") | 164693 |
| S25 | (MH "Randomized Controlled Trials+") | 94508 |
| S26 | TI ("randomized controlled trial" or "randomized clinical trial" or "controlled clinical trial" or "randomized") OR AB ("randomized controlled trial" or "randomized clinical trial" or "controlled clinical trial" or "randomized") OR SU ("randomized controlled trial" or "randomized clinical trial" or "controlled clinical trial" or "randomized") | 215230 |
| S27 | S1 OR S2 | 28050 |
| S28 | S3 OR S4 OR S5 OR S6 OR S7 OR S8 OR S9 OR S10 OR S11 OR S12 OR S13 OR S14 OR S15 OR S16 OR S17 OR S18 OR S19 OR S20 OR S21 OR S22 OR S23 OR S24 | 327515 |
| S29 | S25 OR S26 | 215239 |
| S30 | S27 AND S28 AND S29 | 255 |

**WOS**

| Search | Query | Results |
| --- | --- | --- |
| #1 | TS=("mild cognitive impairment*" or "cognitive dysfunction*" or "cognitive defect" or "cognitive declin*" or "cognitive impairment*" or "mild neurocognitive disorder*" or "mental deterioration*" or "age associated cognitive decline" or "pre-dementia") | 216567 |
| #2 | TS=("dietary supplement*" or "nutrition therapy" or "nutritional supplement*" or "nutrition*" or "nutrient") OR TS=("vitamin A" or "VA" or "vitamin B" or "VB" or "folic acid" or "FA" or "Vitamin B9" or "B9" or "Vitamin B6" or "B6" or "Vitamin B12" or "B12" or "antioxidant* " or "vitamin C" or "VC" or "vitamin D" or "VD" or "vitamin E" or "VE" or "vitamin K" or "VK" or "fatty acid*" or "PUFA" or "omega 3 fatty acid*" or "docosahexaenoic acid" or "DHA" or "α-Linolenic acid" or "ALA" or "eicosapentaenoic acid" or "EPA" or "omega 6 fatty acid*" or "arachidonic acid" or "AA" or "linoleic acid" or "LA" or "γ- Linolenic acid" or "GLA") | 5533994 |
| #3 | TS=("randomized controlled trial" or "randomized clinical trial" or "controlled clinical trial" or "randomized") | 1154185 |
| #4 | #1 AND #2 AND #3 | 1753 |

**CNKI**

| Search | Query | Results |
| --- | --- | --- |
| #1 | SU=('mild cognitive impairment' + 'cognitive dysfunction' + 'cognitive defect') and SU=('dietary supplement' + 'nutrition therapy' + 'nutritional supplement' + 'nutrition' + 'nutrient' + ‘vitamin A’ + 'vitamin B' + ‘vitamin C’ + ‘vitamin D’ + ‘vitamin E’ + ‘vitamin K’ + 'antioxidant ' + 'fatty acid' + ‘docosahexaenoic acid’ +‘eicosapentaenoic acid’) and SU=('randomized controlled trial' + 'randomized clinical trial' + 'controlled clinical trial') | 16 |

**WangFang**

| Search | Query | Results |
| --- | --- | --- |
| #1 | SU:("mild cognitive impairment" or "cognitive dysfunction" or "cognitive defect") and SU:( "dietary supplement" or "nutrition therapy" or "nutritional supplement" or "nutrition" or "nutrient" or "vitamin A" or "vitamin B" or "vitamin C" or "vitamin D" or "vitamin E" or "vitamin K" or "antioxidant" or "fatty acid" or "docosahexaenoic acid" or "eicosapentaenoic acid") and SU:("randomized controlled trial" or "randomized clinical trial" or "controlled clinical trial") | 34 |

**SinoMed**

| Search | Query | Results |
| --- | --- | --- |
| #1 | "mild cognitive impairment"[SU] | 7 |
| #2 | "mild cognitive impairment" OR "cognitive dysfunction" OR "cognitive defect" | 32189 |
| #3 | "vitamin A"[SU] OR "vitamin B"[SU] OR "vitamin C"[SU] OR "vitamin D"[SU] OR "vitamin E"[SU] OR "vitamin K"[SU] OR "antioxidant"[SU] OR "fatty acid"[SU] OR " docosahexaenoic acid "[SU] OR " eicosapentaenoic acid "[SU] | 156428 |
| #4 | "dietary supplement" OR "nutrition therapy" OR "nutritional supplement" OR "nutrient" | 63187 |
| #5 | "randomized controlled trial"[SU] | 495181 |
| #6 | "randomized controlled trial" OR "randomized clinical trial" OR "controlled clinical trial" | 518263 |
| #7 | #1 OR #2 | 32189 |
| #8 | #3 OR #4 | 218713 |
| #9 | #5 OR #6 | 518185 |
| #10 | #7 AND #8 AND #9 | 22 |

**Supplemental Table S2. Limitations of Included Studies.**

| **Study** | **Limitations** |
| --- | --- |
| Mengelberg et al. (2022) | - Incomplete baseline blood concentrations of nutrients - No widely applied cognition scale - Small sample size |
| Perła-Kaján et al. (2021) | - No baseline blood concentrations of nutrients |
| Li et al. (2021) | - Small sample size - Short duration of intervention |
| Bai et al. (2021) | - Small sample size - Short duration of intervention |
| Yang et al. (2020) | —— |
| Stavrinou et al. (2020) | - No baseline blood concentrations of nutrients - Small sample size - Short duration of intervention |
| Ma, Li et al. (2019) | —— |
| Ma, Zhou et al. (2019) | - Short duration of intervention |
| Fan et al. (2017) | - No baseline blood concentrations of nutrients - Short duration of intervention |
| Zhang et al. (2017) | - No widely applied cognition scale |
| Bo et al. (2017） | - Short duration of intervention |
| Naeini et al. (2014) | —— |
| Lee et al. (2013) | - Small sample size |
| de Jager et al. (2012) | - No widely applied cognition scale |

**Supplemental Figure S1. Subgroup analysis on global cognitive function according to** **trial region (China region, other regions).**

**
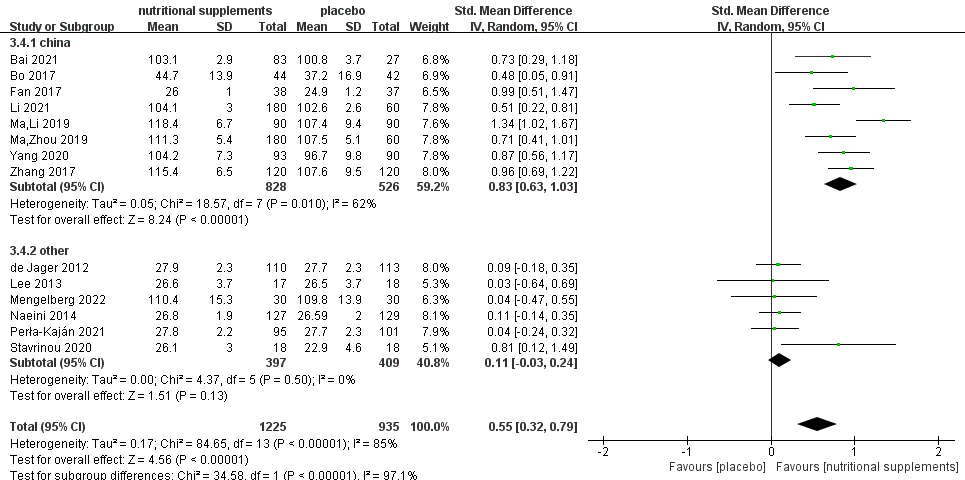
**

**Supplemental Figure S2. Subgroup analysis on global cognitive function according to number of each group (N<50, N>50).**


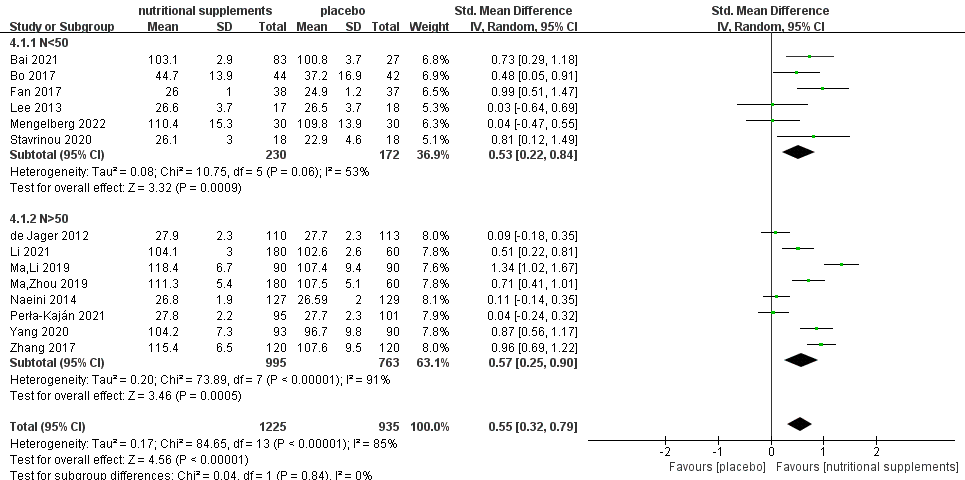


**Supplemental Figure S3. Subgroup analysis on global cognitive function according to age of participants (age<70, age>70).**


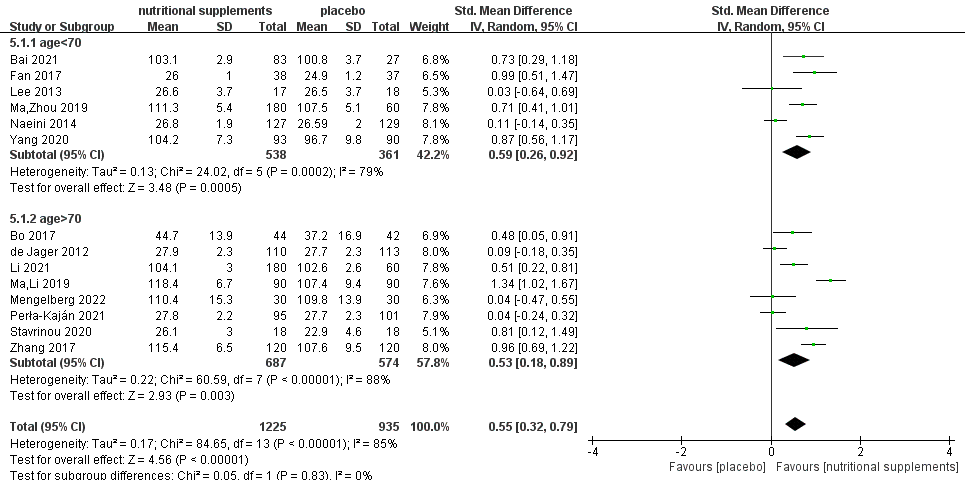


**Supplemental Figure S4. Meta-regression analysis on global cognitive function according to number of each group (N<50, N>50).**


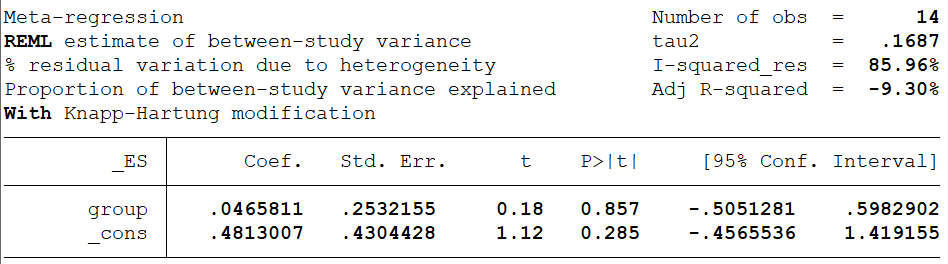


**Supplemental Figure S5. Meta-regression analysis on global cognitive function according to age of participants (age<70, age>70).**


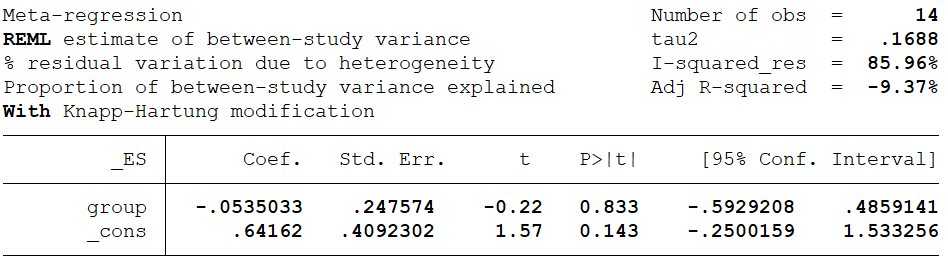


**Supplemental Figure S6. Meta-regression analysis on global cognitive function according to** **durations (6 months, 12 months, 24 months).**


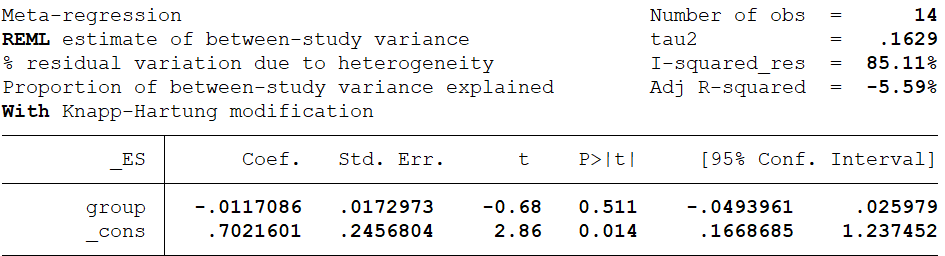


**Supplemental Figure S7. Effect of vitamins and PUFAs on visuospatial skills.**

**
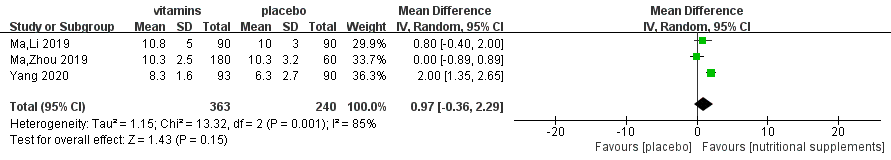

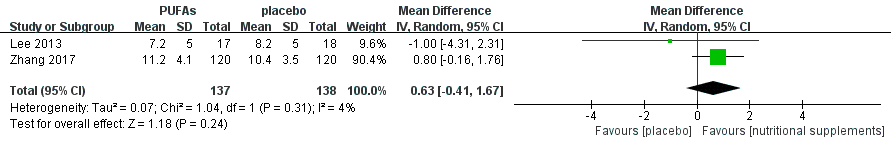
**

**Supplemental Figure S8. Effect of vitamins and PUFAs on executive function.**

**
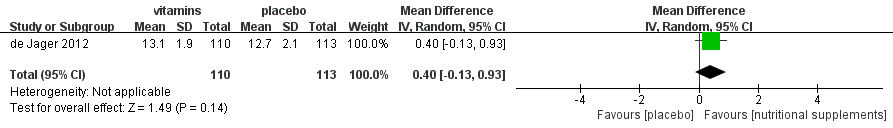

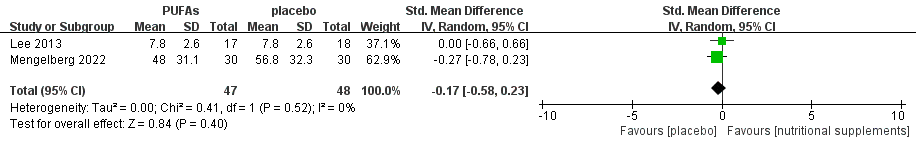
**

**Supplemental Figure S9. Effect of vitamins and PUFAs on processing speed.**

**
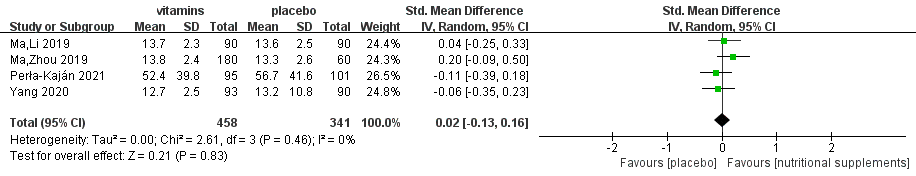

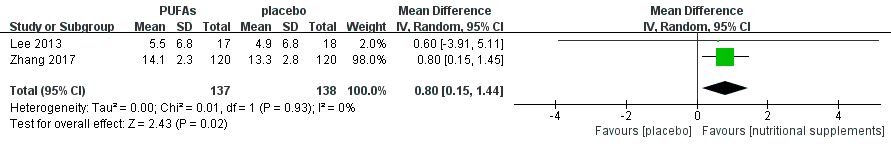
**
